# AgentBasedModeling.jl: A tool for stochastic simulation of structured population dynamics

**DOI:** 10.1371/journal.pcbi.1014366

**Published:** 2026-07-29

**Authors:** Paul Piho, Philipp Thomas

**Affiliations:** Department of Mathematics, Imperial College London, London, United Kingdom; University of California Irvine, UNITED STATES OF AMERICA

## Abstract

Agent-based modeling is a powerful approach for understanding cellular systems. Yet, many existing frameworks treat cell-level and population behaviors separately, overlooking their interplay. We present AgentBasedModeling.jl, a Julia package for simulating stochastic, continuous-time agent-based models that integrate intracellular processes with population dynamics. In our stochastic framework, agents evolve according to general continuous-time jump-diffusion dynamics and interact via continuous-rate jump processes. It supports flexible specification of the underlying measure-valued process of intracellular reaction networks and cell growth, capturing events such as cell division, death, intercellular communication, and environmental interactions. We demonstrate the use of the package by validating it on models of stochastic gene expression in growing cells and providing new insights into cell-cell communication and stochastic phage infection. Our tool provides a flexible and efficient platform for exploring how single-cell stochasticity drives emergent population-level behaviors in structured biological systems.

## 1. Introduction

Interacting autonomous agents, such as living cells, can exhibit complex dynamics requiring extensive computer simulation. The diversity and emergent phenomena observed in cell populations arise from the interactions among agents with heterogeneous characteristics, known as population structure. Such structures present states that manifest in the composition of cells, including DNA, proteins, metabolites, and the overall cell physiology, which encompasses cell age, size, and growth rate, all of which are responsive to external signals and subject to internal variations. Agent-based approaches in cellular systems range from gene expression [1–4] to cell populations, multi-cellular organisms [5], cancer [6–9] and immune cell interactions, and extend to complex systems such as individual-based models of epidemics [10–13].

Simulation methods for cellular systems are gaining momentum as more data combine with computing power, for example, to enable simulation-based inferences [14–17]. While many tools exist for constructing and simulating agent-based models, such as *Agents.jl* [18], MESA [19], Repast Simphony [20] and NetLogo [21], these tools are often geared towards spatial structure. Their limitation lies in their focus on incremental, time-stepped progression or probabilistically distributed event times, which require cellular states not to change between events, as assumed in Gillespie’s algorithm [22]. Living cells rarely meet such conditions. When cell states are dynamic, these methods do not sample the underlying stochastic process exactly and are thus not universally valid for cellular population dynamics.

Existing approaches overcome these limitations by discretizing state evolution into a series of steps or pseudo-compartments [23–26]. These approaches introduce additional network complexity and approximation errors that can be difficult to control in practice. In the context of structured cell population dynamics that describe agents with different ages, life cycles, genetic differences, or biochemical makeups [27], exact approaches that employ continuous-rate formulations of agent-based systems remain limited [28,29]. Several established cell-based agent-based frameworks, like BSim [30], PhysiCell [31], and Chaste [32], support hybrid models for simulating cells with internal state dynamics governed by ordinary differential equations (ODEs), embedded within spatial environments that include physical constraints and diffusible signals. These tools are particularly suited to modeling bacterial populations, multicellular development, and tumors.

Jump-diffusion processes [33] provide a generic continuous-rate framework for modeling stochastic cellular dynamics implemented in standard packages such as *JumpProcesses.jl* [34] or simulators of individual cells like *SGNS2* [28] and *PyEcoLib* [29]. These tools have not yet been applied to structured cell populations, which have traditionally been described by deterministic partial differential equations [35] or measure-valued stochastic processes [36,37]. In the agent-based world, such modeling considerations have led to the development of tailored frameworks, such as multi-scale models for cell populations [7,30,38], where the intracellular dynamics of cells evolve continuously, specified by ordinary differential equations. However, general and extensible tools for structured populations described by measure-valued stochastic processes, which couple stochastic state dynamics of interacting agents in continuous time, are still limited.

Here, we introduce *AgentBasedModeling.jl*, which enables easy specification and simulation of stochastic agent-based cell models where internal agent dynamics are modeled as jump-diffusion processes and influence population-level interactions (illustrated in Fig 1). We implement an exact simulation algorithm, allowing the rates of interactions between agents to depend on the continuously evolving internal states of the agents. Our tool integrates easily with the existing mathematical modeling tools of *ModelingToolkit.jl* [39] for differential equations and *Catalyst.jl* [40] for stochastic reaction networks, thus providing a flexible and scalable modeling framework for structured cell populations in *Julia*.

**Fig 1:**
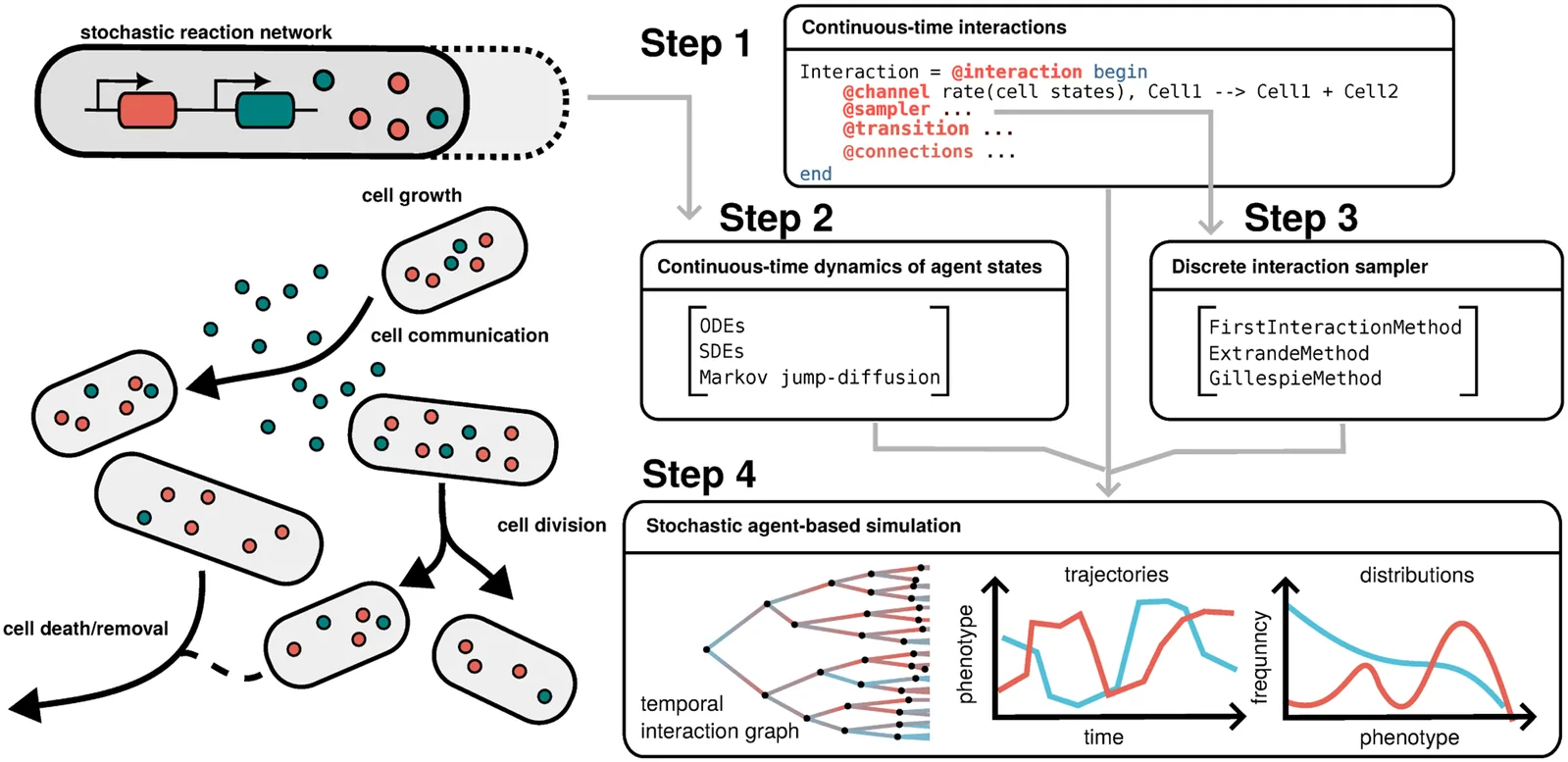
Overview of the AgentBasedModeling.jl workflow. Agent-based models of cells interacting via cell-cell communication, cell division or death. Cell states may arise from the coupling of intracellular reaction networks, cell growth and other modeled factors (left). The package combines cell interactions in continuous time (STEP 1) with exact stochastic sampling of the cells’ state dynamics and temporal interactions (STEP 2 and 3) and outputs temporal graphs, trajectories or distributions (STEP 4).

## 2. Design and implementation

### 2.1. A framework for agent-based modeling of structured cell populations

We consider interactions between agents to depend on the agent type and their internal states that evolve continuously over time. With the $i^{th}$ agent we associate an agent type $S_{j}$, where j∈1,...,M, and a state vector ${𝐱}_{i}(t)$, which is a realization of a Markov jump-diffusion process:


$$\begin{array}{c} d{𝐱}_{i}(t)={𝐟}_{j}({𝐱}_{i}(t),t)dt+\sigma_{j}({𝐱}_{i}(t),t)d{𝐖}_{i}(t)+\sum_{r}\nu_{rj}dY_{ri}(t), \end{array}$$ (1)

where ${𝐟}_{j}$ is a deterministic drift vector corresponding to ordinary differential equation (ODE); $\sigma_{j}({𝐱}_{i}(t))d{𝐖}_{i}(t)$ is state-dependent Gaussian white noise corresponding to the stochastic differential equation part (SDE); and $dY_{ri}$ are Poisson process jumps with path-dependent intensity $\int_{0}^{t}dsw_{rj}({𝐱}_{i}(s))$ and increments $\nu_{rj}$ that define the jump process of agent type $S_{j}$.

We define an interaction channel *c* that takes $k_{c}$ input agents of types $S_{n_{1}^{c}},...,S_{n_{k_{c}}^{c}}$ with internal states ${𝐱}_{1}^{-},...,{𝐱}_{k_{c}}^{-}$, which we denote as $S_{n_{1}^{c}}[{𝐱}_{1}^{-}],...,S_{n_{k_{c}}^{c}}[{𝐱}_{k_{c}}^{-}]$, and creates $l_{c}$ output agents of types $S_{m_{1}^{c}},...,S_{m_{l_{c}}^{c}}$ with states ${𝐱}_{1}^{+},...{𝐱}_{l_{c}}^{+}$:


$$\begin{array}{cc} & S_{n_{1}^{c}}[{𝐱}_{1}^{-}]+...+S_{n_{k_{c}}^{c}}[{𝐱}_{k_{c}}^{-}]\overset{r_{c}({𝐱}_{1}^{-},...,{𝐱}_{k_{c}}^{-},t)\;}{\to }S_{m_{1}^{c}}[{𝐱}_{1}^{+}]+...+S_{m_{l_{c}}^{c}}[{𝐱}_{l_{c}}^{+}]\\ & {𝐱}_{1}^{+},...,{𝐱}_{l_{c}}^{+}\sim B_{c}(•|{𝐱}_{1}^{-},...,{𝐱}_{k_{c}}^{-}), \end{array}$$ (2)

where $n_{1}^{c},...,n_{k_{c}}^{c},m_{1}^{c},...,m_{l_{c}}^{c}\in \{1,...,M\}$ are the indices of agent types involved in the interaction. The rate function $r_{c}$ of the rule is computed using the internal states of the input agents ${𝐱}_{1}^{-},...,{𝐱}_{k_{c}}^{-}$. The states of the output agents ${𝐱}_{1}^{+},...{𝐱}_{l_{c}}^{+}$ are initialized probabilistically according to the transition kernel $B_{c}$ that defines the conditional probability of creating output agent states ${𝐱}_{1}^{+},...{𝐱}_{l_{c}}^{+}$ given the input agent states ${𝐱}_{1}^{-},...{𝐱}_{k_{c}}^{-}$.

In simple terms, an interaction channel represents a reaction or rule, analogous to the definition of reactions in biochemical kinetics. However, here the agents’ internal states modulate both the interaction rate function and the transition kernel. Thus, mapping a channel to a set of reactions amounts to enumerating all possible agent states, potentially yielding an infinite number of reactions. This map is effectively expressed through the interaction channel analogous to rule-based frameworks [23,41,42]. For our purpose, we consider cells as agents whose populations comprise different cell types. For example, we can describe asymmetric stem cell division of a stem cell *S*_1_ differentiating towards *S*_2_, via the rule $S_{1}[{𝐱}_{1}^{-}]\overset{r_{c}({𝐱}_{1}^{-})\;}{\to }S_{1}[{𝐱}_{1}^{+}]+S_{2}[{𝐱}_{2}^{+}]$, which could depend on the cell cycle position ${𝐱}_{1}^{-}$ of the mother cell. Similarly, we could consider cell-cell interaction via rules such as $S_{1}[{𝐱}_{1}^{-}]+S_{2}[{𝐱}_{2}^{-}]\overset{r_{c}({𝐱}_{1}^{-},{𝐱}_{2}^{-})\;}{\to }S_{1}[{𝐱}_{1}^{+}]+S_{2}[{𝐱}_{2}^{+}]$, where ${𝐱}_{1,2}^{-}$ encode ligand-receptor interactions and $\left({𝐱}_{1}^{+},{𝐱}_{2}^{+})\sim B_{c}(\cdot |{𝐱}_{1}^{-},{𝐱}_{2}^{-}\right)$ encodes the resulting transcriptional changes.

### 2.2. *AgentBasedModeling.jl* facilitates model specification and integrates easily with deterministic and stochastic modeling tools

*AgentBasedModeling.jl* package allows for convenient model specification and simulation of such models. The first step (Fig 1 Step 1) in the specification concerns the definition of interaction channels (Eq 2). These are implemented with the @interaction environment with the lines of code following the specification:


$$\begin{array}{cc} & \begin{array}{cc} @channel\;r_{c}({𝐱}_{1}^{-},...,{𝐱}_{k_{c}}^{-},t),\; & S_{n_{1}^{c}}+...+S_{n_{k_{c}}^{c}}\to S_{m_{1}^{c}}+...+S_{m_{l_{c}}^{c}} \end{array}\\ & \begin{array}{cc} @connection\;( & {𝐱}_{1}^{-}\Rightarrow {𝐱}_{1}(t),...,{𝐱}_{k_{c}}^{-}\Rightarrow {𝐱}_{k_{c}}(t)) \end{array}\\ & \begin{array}{cc} @transition\;( & {𝐱}_{1}(t)\Rightarrow {𝐱}_{1}^{+},...,{𝐱}_{l_{c}}(t)\Rightarrow {𝐱}_{l_{c}}^{+}) \end{array} \end{array}$$

where ${𝐱}_{1}^{+},...,{𝐱}_{l_{c}}^{+}$ are sampled from the transition kernel $B_{c}(•|{𝐱}_{1}^{-},...,{𝐱}_{k_{c}}^{-})$. The @channel line defines the rate function $r_{c}$ of the interaction and the interaction stoichiometry while the @transition line defines the initializations of the output agents given the transition kernel *B*. The @connections line is used to indicate which traits of which agents correspond to the symbols used in the expressions. The tuple $\left({𝐱}_{1}^{-}\Rightarrow {𝐱}_{1}(t)\right)$ denotes that the value of vector ${𝐱}_{1}^{-}$ corresponds to the state ${𝐱}_{1}(t)$ of the first input agent at time *t*. These rules are matched to the individual instances of agents in the simulation.

The second step (Fig 1, Step 2) in the specification is to define the internal state dynamics for all agent types (Eq 1). This is done as an ODE, SDE, jump process, or a combination of them using *ModelingToolkit.jl* [39] and *Catalyst.jl* [40], leveraging the existing *Julia* programming language ecosystem.

### 2.3. *AgentBasedModeling.jl* enables exact simulation of stochastic agent-based models

*AgentBasedModeling.jl* provides a stochastic simulation algorithm to exactly simulate any agent-based model. The outline of the algorithm is given in Algorithm 1 and is based on the first reaction method for simulating Markov jump processes [43]. To sample the next interaction time in a simulation time-interval [t,t+Δt], the algorithm computes the trajectories of agent states in that interval. This is dependent on the model given for the agent state dynamics and can, for example, involve solving a system of ODEs, SDEs, or sampling a trajectory of a jump-diffusion process for each agent in the population. We then construct a set of possible interaction instances between agents for each interaction channel. For each channel, the simulation algorithm samples and executes the instance with the fastest interaction time.

**Algorithm 1. Stochastic simulator for the population models**.

**Require:** Simulation time-span [*T*_0_, *T*], lookup horizon Δt.

Let $t\leftarrow T_{0}$ and $t_{w}\leftarrow T_{0}+\Delta t$ and consider the time window $\left[t,t_{w}\right]$.

Let 𝒮 be the initial population of agents at time *t* defined by pairs of agent types and sta*t*es at time *t*.

For all agents in 𝒮 simulate the state trajectories in the window $\left[t,t_{w}\right]$.

Let *C* be a set of interaction channels.

**while** t≤T **do**

For each c∈C with input agent types $S_{n_{1}^{c}},\cdots ,S_{n_{k_{c}}^{c}}$ construct the set $A_{c}$ of all without replacement combinations of agents in the population 𝒮 that match the input types.

For all channels *c* let $f_{c}(a,s)$ be the interaction rate for a combination of agents *a* at time $s\in [t,t_{w}]$ and let ${\bar{f}}_{c}(a)$ denote the upper bound of an interaction rate for all $s\in [t,t_{w}]$. Utilize one of the following algorithms: the first reaction method where for each $a\in A_{c}$ use the thinning algorithm [44] with rate $f_{c}(a,s)$ and bound ${\bar{f}}_{c}(a)$ to sample next interaction times $t_{a}\in [t,t_{w}]$ and choose the time $t_{c}$ and input agents *a* corresponding to ${argmin}_{a}t_{a}$; or apply the Extrande algorithm [45] with rate bound $\sum_{a\in A_{c}}{\bar{f}}_{c}(a)$ to sample an interaction time $t_{c}$ and the input agents *a*.

Find the channel *c* with the least next interaction time $\hat{t}={min}_{c}t_{c}$ and the corresponding combination of input agents $a_{c}$.

**if** $\hat{t}\leq t_{w}$ **then**

Given the set of input agents $a_{c}\subset 𝒮$ with states ${𝐱}_{n_{1}^{c}}(\hat{t}),...,{𝐱}_{n_{k_{c}}^{c}}(\hat{t})$ at time $\hat{t}$ and output agent types $S_{m_{1}^{c}},...,S_{m_{l_{c}}^{c}}$, create output agents with the given types and states ${𝐱}_{m_{1}^{c}}^{+},...,{𝐱}_{m_{l_{c}}^{c}}^{+}$ at time $\hat{t}$ sampled from the kernel $B_{c}(•|{𝐱}_{n_{1}^{c}}(\hat{t}),...,{𝐱}_{n_{k_{c}}^{c}}(\hat{t}))$. Remove input agents $a_{c}$ from 𝒮.

Simulate the state trajectories of the output agents in the time interval $\left[\hat{t},t_{w}\right]$, add the output agents to 𝒮, and let $t\leftarrow \hat{t}$.

**else**

Let $t\leftarrow t_{w}$, $t_{w}\leftarrow t+\Delta t$ and simulate the state trajectories of all agents in 𝒮 in time window $\left[t,t_{w}\right]$.

**end if**

**end while**

The package offers three algorithms for sampling the next interaction instance of a given channel, and different algorithms can be combined within a simulation model for each channel (Fig 1 Step 3). Simulation algorithms are specified within the @interaction keyword using the @sampler keyword. The first method FirstInteractionMethod() samples interaction times for each instance of an interaction using the thinning algorithm [44]. The second algorithm uses the Extrande method [45] to sample the next interaction time for each interaction channel and can be used by specifying ExtrandeMethod(...). An alternative but potentially unchecked approximation to these aforementioned methods is the GillespieMethod().

The algorithms FirstInteractionMethod(bfn, L) and ExtrandeMethod(bfn, L) require specifying a symbolic function bfn returning a constant upper bound in the time interval [*t*, *t* + *L*], where *L* is a user-specified lookup time horizon. For convenience, the bound can be computed automatically using the optional boundtype keyword, e.g., ExtrandeMethod(L; boundtype = :unknown), which can assume:increasing,:decreasing or:unknown values. In these cases, the interaction rate function is bounded by assuming monotonically increasing or decreasing functions of time, i.e., the bound in a time interval [*t*, *t* + *L*] is the interaction rate at time *t* + *L* or *t*, respectively. When the keyword argument is:unknown, which is the default setting, the algorithm computes the upper bound of the interaction rate by evaluating the rate at each timestep in the interval [*t*, *t* + *L*] produced by simulating the agent state dynamics and taking the maximum of the computed rates on the fly.

Although our simulation methods are rooted in Markovian dynamics, state-dependent interaction rates generate effective time-dependent event rates, leading to non-exponential waiting-time distributions even when the full process remains Markovian in an appropriately extended state space. In *AgentBasedModeling.jl*, such dynamics can be represented by augmenting agents with internal state variables that modulate event propensities. This provides a natural framework for modelling history-dependent cellular events, such as size-dependent cell division or infection-age-dependent lysis events (see Section 3).

### 2.4. *AgentBasedModeling.jl* provides tailored simulation outputs

Agents and their states can be tracked at individual and population levels (Fig 1, Step 4). We distinguish (i) snapshot and (ii) trajectory simulation, (iii) interaction snapshots, and (iv) temporal interaction networks, which we will describe below. Biological data examples of snapshot measurements include cell size, growth, or gene expression obtained from smFISH, flow cytometry, single-cell RNA sequencing, or multi-omics technologies. Trajectories present common measurements in population dynamics such as the microbiome, cancer growth, infections, or drug responses. Interaction snapshots represent agent states sampled at interaction events, such as cell birth, division, or cell-cell interactions. Temporal interaction networks are the most complex output, enabling the tracking of cellular agents and states, such as those observed in time-lapse microscopy or lineage tracing [46]. The handling of these outputs in the package is described below.

A simulation is processed by specifying a model, a set of parameters, and an initial condition. The simplest output is a snapshot of the state of the population at the simulation endpoint, which we denote as a **snapshot simulation**:


$$\begin{array}{cc} & result=simulate(model,init,params)\\ & result.final \end{array}$$

which requires specification of a model, initial conditions init and parameters params. Furthermore, the package can provide **trajectory simulation** representing a series of population snapshots at equidistant time points. These can represent counts of agents and types or snapshots across the agents’ states. For example, for an agent *A* with state *s* this can be obtained through using


$$\begin{array}{c} params=SimulationParameters(...,\Delta \;t,...;\;snapshot=[PopulationSnapshot(A),StateSnapshot(A,s),...]) \end{array}$$

where PopulationSnapshot(A) counts agents of type *A* and StateSnapshot(A, s) provides a snapshot of the agent state every timestep Δ*t*.

Other outputs concern **interaction snapshots** that track states of the input and output agents of specified interactions when events occur. These can be accessed by selecting the states in the @interaction environment using @saveinstate and @saveoutstate Keywords:


$$\begin{array}{cc} & \begin{array}{c} @saveinstate\;(S_{n_{1}},x_{1})\;(S_{n_{2}},x_{2})\;...(S_{n_{k}},x_{k}) \end{array}\\ & \begin{array}{c} @saveoutstate\;(S_{m_{1}},x_{1})\;(S_{m_{2}},x_{2})\;...(S_{m_{l}},x_{l}) \end{array} \end{array}$$

Finally, the most complex output is a **temporal interaction graph** that represents a function-valued directed graph whose edges represent trajectories of agent states and vertices of sampled interactions between these agents:


$$\begin{array}{cc} & vertices=result.interactions\\ & edges=result.agents. \end{array}$$

As the tool is implemented as a package for the *Julia* language, we can utilize existing statistical and visualization packages for analyses. For example, visualisation of such a graph can be conveniently implemented using *MetaGraphsNext.jl* from the *Graphs.jl* ecosystem.

## 3. Results

We demonstrate the use and functionality of *AgentBasedModeling.jl* with three examples of varying complexity. We first provide a hands-on, step-by-step example of gene expression model with a simple interaction structure and complex state models, coupling intracellular reaction networks with cell size, growth and cell division. We then investigate how cell-to-cell communication with feedback can lead to population-level synchronous state switching. Finally, we investigate the dynamics of infection-lysis by bacteriophages as a stochastic extinction event.

### 3.1. Stochastic gene expression is coupled to growth and division

We consider a stochastic agent-based model of gene expression coupled to cell growth (Fig 2a) using a combination of *ModelingToolkit.jl*, *Catalyst.jl* and *AgentBasedModeling.jl* (S1 Code). Such models explain concentration homeostasis [47–49] and account for extrinsic noise in cells [16,50,51]. We first consider the intracellular dynamics of cells whose state is given by cell size *s*, added size Δ, and protein count *p* that are specified using the @abm_variables keyword. Cell size *s* increases deterministically with exponential growth rate α and can be modeled by an ODE. Proteins *p* are expressed in geometrically distributed bursts *m* modeled by a Markov jump process. The @reaction_network keyword provided by *Catalyst.jl* combines these dynamics, which are then passed to AgentDynamics structure from *AgentBasedModeling.jl* as shown in Step 1.

**Fig 2:**
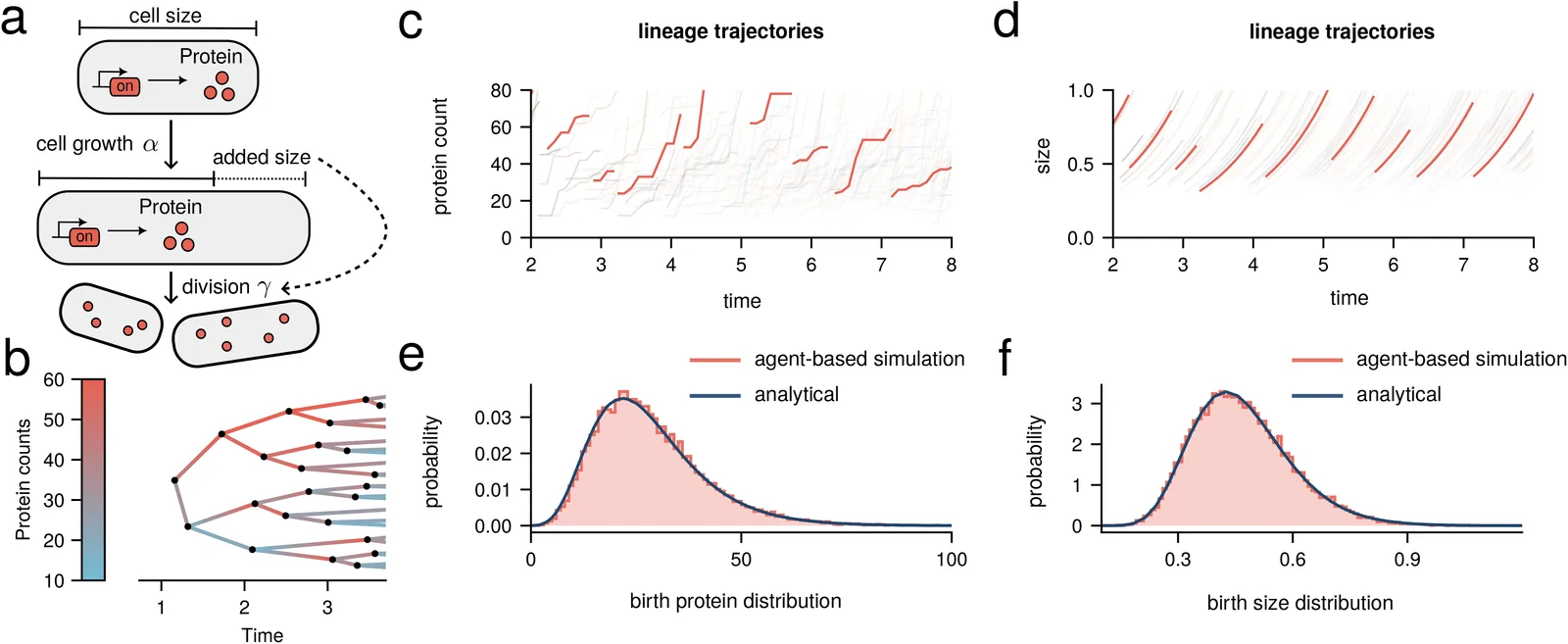
Stochastic gene expression is coupled to cell size in growing and dividing cells. **(a)** Cartoon of the model involving bursty protein expression in growing and dividing cells with growth rate α and division rate γ. Molecules are stochastically partitioned at cell division. **(b)** Lineage tree of growing cell population with protein levels superimposed. **(c-d)** Simulation of protein content and cell size for a lineage tree (shaded). A representative single cell tracked over several divisions is highlighted (red). **(e-f)** Simulated birth protein and size distributions at cell birth across lineage trees (red) are compared with the analytical computations (black).

**Step 1: Stochastic gene expression with cell division and growth**



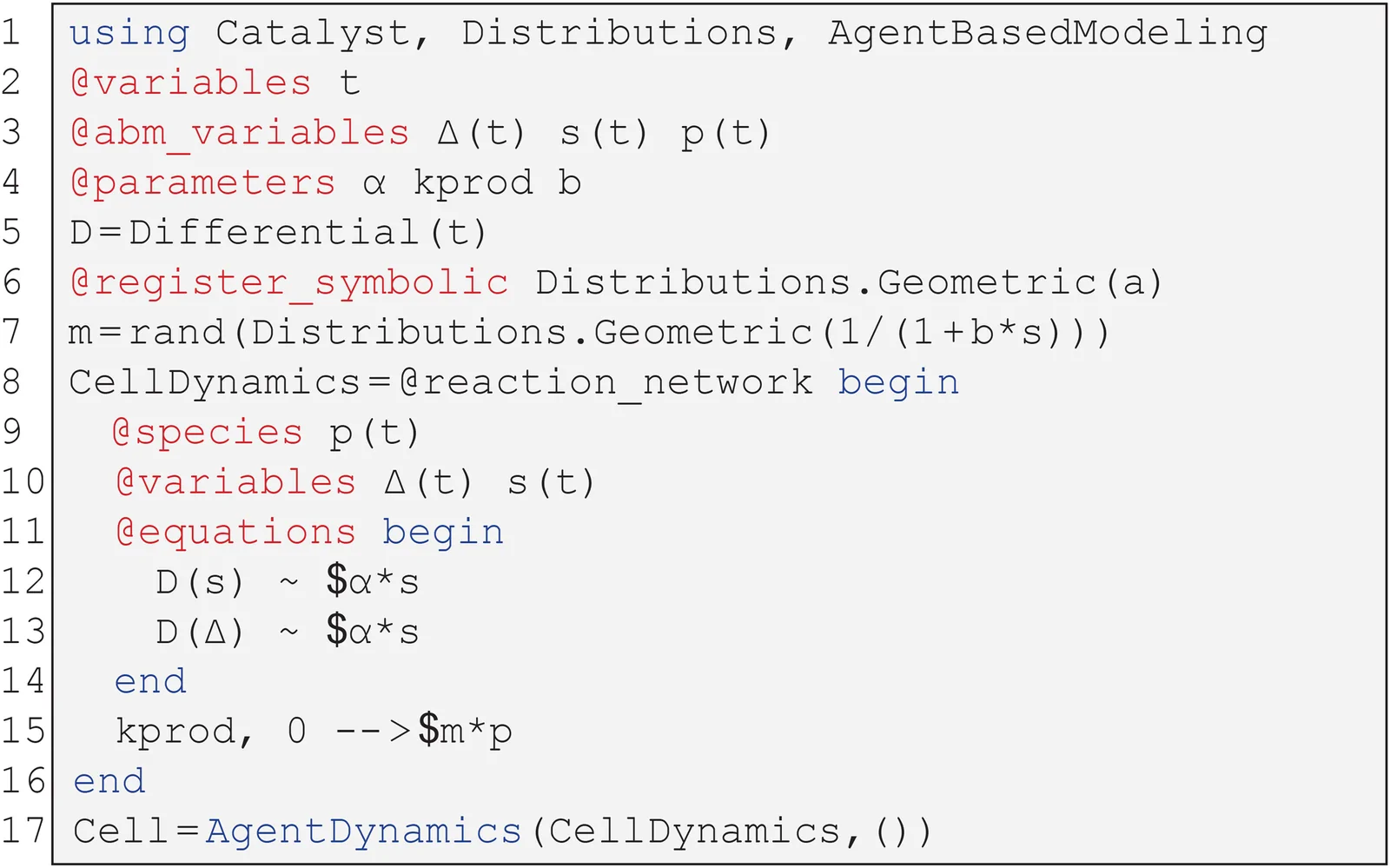



Cells divide via the adder mechanism, where a stochastic size increment is added between divisions, as observed in many cell types. In Step 2, we implement the interaction channel via the @interaction keyword as follows. First, we assign variables Δ, *s*, *p* to the agent states via the @connections keyword. The interaction channel can be written as


$$\begin{array}{cc} & \text{Cell}[\Delta ,s,p]\overset{\gamma_{\text{division}}(s,\Delta ,\alpha )\;}{\to }\text{Cell}[0,s_{1}^{+},p_{1}^{+}]+\text{Cell}[0,s_{2}^{+},p_{2}^{+}], \end{array}$$ (3)

$$\begin{array}{cc} & s_{1}^{+}\sim \beta s,\;s_{2}^{+}\sim (1-\beta )s,\;p_{1}^{+}\sim \text{Binomial}(\beta ,p),\;p_{2}^{+}\sim p-p_{1}^{+}. \end{array}$$ (4)

with a size-dependent division rate


$$\begin{array}{c} \gamma_{\text{division}}(s,\Delta ,\alpha )=\underset{\text{growth}}{\underbrace{\alpha s}}\;\underset{\text{cell size control}}{\underbrace{\gamma_{\text{size}}(\Delta )}}, \end{array}$$ (5)

where $\gamma_{\text{size}}(\Delta )$ denotes the cell size control (see S1 Code) and β~Beta() denotes variability in the placement of the division site [52]. The keyword @channel specifies the channel stoichiometry and the division rate. During cell division, cells are split, and proteins are partitioned equally between the two daughter cells. The transition kernel $B(s_{1},p_{1},s_{2},p_{2}|s,p)$ is sampled from beta and binomial distributions within the @transition keyword. It defines the probability of the two daughter cells inheriting sizes $s_{1},s_{2}$ and protein counts $p_{1}$ and $p_{2}$ respectively, given the mother cell divides with size *s* and protein count *p*. We sample this interaction channel with the Extrande method specified via the @sampler keyword. For convenience, the optional keyword boundtype = :increasing computes the bound automatically, assuming the division rate and cell size are monotonically increasing with time. The keywords @saveinstate and @saveoutstate record the cell states of cell size *s* and protein levels *p* before and after division for analysis.

**Step 2: Stochastic gene expression with cell division and growth**



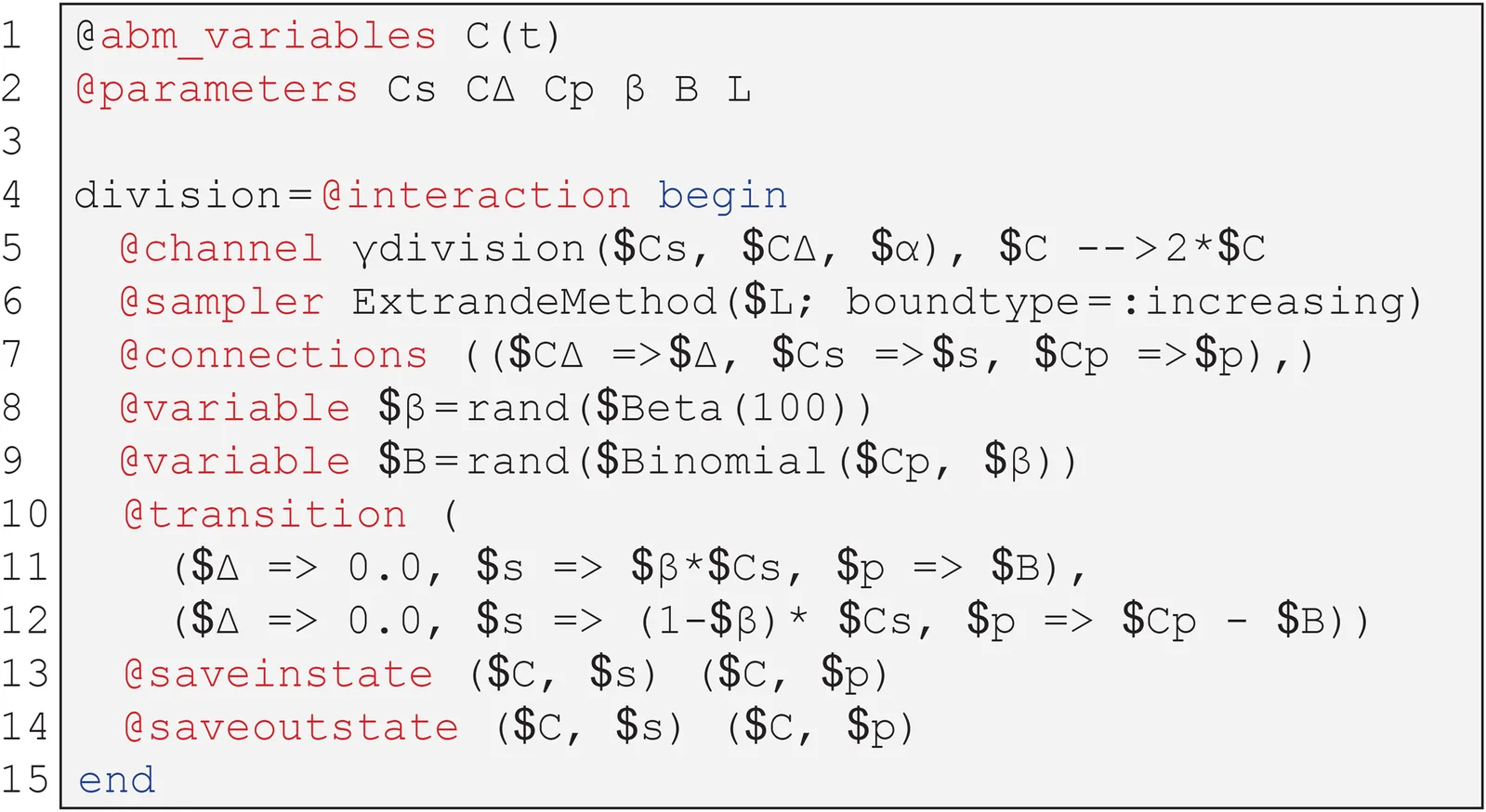



Finally, in Step 3, the interactions and state dynamics are composed into a simulation model with the AgentsModel function. The function simulate() performs the simulation with parameters passed by SimulationParameters().

**Step 3: Stochastic gene expression with cell division and growth**



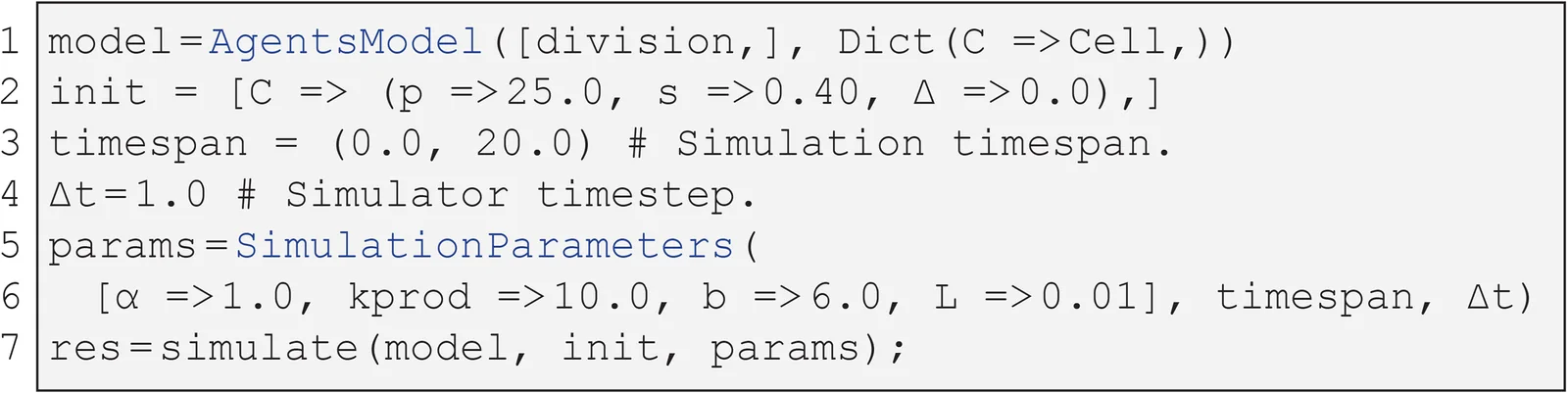



The stochastic simulation performed by *AgentBasedModeling.jl* results in a lineage tree of cells (Fig 2b). Cell state trajectories display growing cell size and protein levels interrupted by stochastic cell division events (Fig 2c-d). We validated our implementation by computing protein distribution and size at cell birth analytically [47,52], which are in excellent agreement with our exact simulation algorithm (Fig 2e-f).

### 3.2. Cell-cell communication enables collective switching

Cell-cell communication regulates interactions between cells from microbial communities to complex multicellular organisms. Bacteria release small, diffusible molecules that enable density-dependent regulatory mechanisms, known as quorum sensing. The process coordinates collective behaviors such as biofilm formation and virulence [53]. Similarly, in multicellular organisms, differentiation is orchestrated by signaling pathways that guide cells toward specific lineages. In disease, dysregulated communication allows tumor cells to hijack normal differentiation cues and reprogram their environment [54,55]. While deterministic models are traditionally used to model these phenotypes [56], these systems lend themselves naturally to agent-based modeling [57,58].

We here explore a general model of communication that includes stochastic effects of cell-cell coupling present in small and finite populations (Fig 3a). The model involves cellular agents that expand exponentially in cell size *s* with rate α and produce inducer molecules *x* whose levels feed back on their production via a Hill-type production rate *f*(*x*,*s*):


$$\begin{array}{c} \emptyset \overset{f(x,s)\;}{\to }x,\;\frac{\partial s}{\partial t}=\alpha s. \end{array}$$ (6)

**Fig 3:**
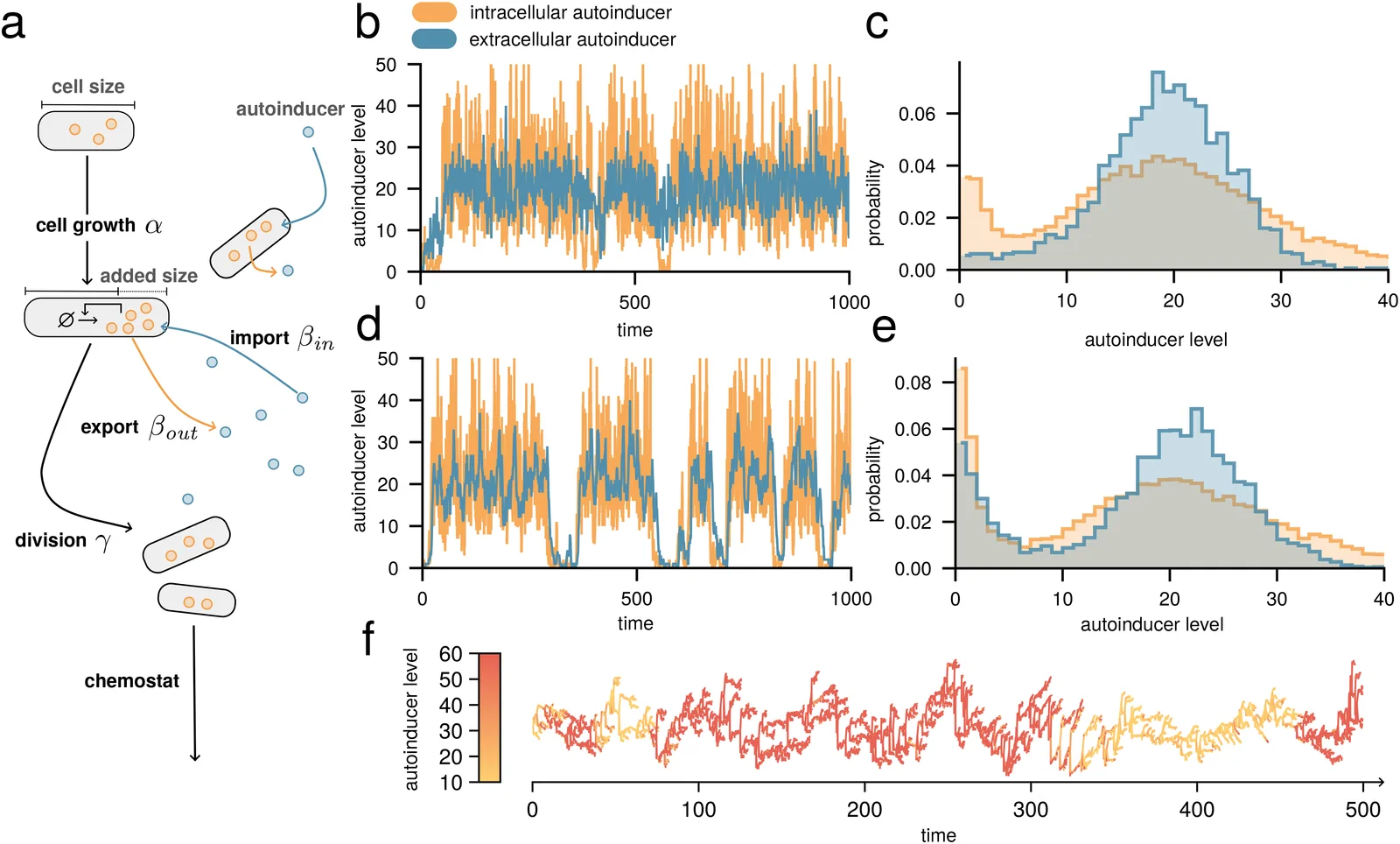
Cell-cell signaling enables collective switching in an agent-based cell model with diffusible autoinducer. **(a)** Illustration of the model involving import and export of an autoinducer molecule produced in cells growing with rate α and dividing with rate γ. We assume competition via a turbidostat, maintaining a constant cell number. **(b)** Trajectories of extracellular and intracellular levels of a random cell traced backwards in time (population of 20 cells). **(c)** Distribution of extracellular signaling molecule is unimodal while intracellular autoinducer shows bimodality. **(d)** Trajectories of extracellular (blue) and intracellular levels (orange) show significant temporal correlations at low population size (5 cells). **(e)** The corresponding distributions of extra- and intracellular autoinducer are bimodal. **(f)** Lineage of a cell population undergoing cell division and dilution in a turbidostat. Superimposed autoinducer level shows synchronised population-level transitions between active and repressed cell states.

The autoinducer is synthesized within cells and then secreted into the extracellular environment, and extracellular molecules *y* can then be sensed and taken up by surrounding cells


$$\begin{array}{c} \text{Env}[y]+\text{Cell}[\Delta ,s,x]\;\overset{\beta }{⇌}\;\text{Env}[y+1]+\text{Cell}[\Delta ,s,x-1]. \end{array}$$ (7)

We further assume that cells grow in microchambers that support a defined population size through dilution at cell division, keeping the population size constant. Once a cell divides, a random cell from the surrounding population is removed to make space for the new daughter cell:


$$\begin{array}{cc} & \text{Cell}[\Delta ,s,x]+\text{Cell}[\cdot ]\overset{\gamma_{\text{division}}(s,\Delta ,\alpha )\;}{\to }\text{Cell}[0,s_{1}^{+},x_{1}^{+}]+\text{Cell}[0,s_{2}^{+},x_{2}^{+}], \end{array}$$ (8)

$$\begin{array}{cc} & s_{1}^{+}\sim \beta s,\;s_{2}^{+}\sim (1-\beta )s,\;x_{1}^{+}\sim \text{Binomial}(\beta ,x),\;x_{2}^{+}\sim x-x_{1}^{+}. \end{array}$$ (9)

Cell division follows the stochastic adder rule and dilutes molecules via binomial partitioning, as before.

We simulated time courses following cells over many cell divisions in the turbidostat (S2 Code). Tracing a cell that was alive at the end of the simulation backward in time, we observed that the environment followed intracellular levels but was generally less variable because it integrates signals from cells (Fig 3b). The distributions of the extracellular autoinducer were unimodal, while the intracellular levels showed bimodal distributions (Fig 3c). This suggests that cells coexist in the inactive and induced states and that the environment follows the mean across cells. Surprisingly, we observed a significant increase in temporal correlations between the extra- and intracellular levels when we decreased the population size in our simulations to what would be appropriate for a mother machine (Fig 3d). Moreover, extracellular levels were bimodal similar to intracellular levels (Fig 3e). The simulations suggested that cells collectively occupy inactive or induced states that reflect low and high environmental inducer states (Fig 3f).

### 3.3. Lineage fate after bacteriophage infection is determined by stochastic phage burst kinetics

Bacteriophage λ is a paradigm for studying cellular decision-making, both at the level of individual cellular pathways and at the level of population dynamics [59]. While these pathways have been described in the manner of Gillespie using detailed stochastic reaction network models [59–62], their population dynamics has been explored using deterministic ODE models [63–66].

We here extend a recently experimentally validated model of infection-lysis dynamics during the lytic cycle of bacteriophage λ [64], incorporating multi-stage infection dynamics and Erlang-distributed lysis timing. Our stochastic model consists of discrete cell agents (Cell) that take up nutrients to grow and divide, and interact with phages dispersed in the environment (Env). The model explicitly accounts for stochasticity in intracellular phage replication, cell growth and division rates. The environment contains nutrients *M* and extracellular phages *P* and a cell’s state consists of the age τ, cell size *s*, intracellular nutrients *N*, and intracellular phages *Q*.

Cells and the environment interact through interaction channels that model phage infection, cell lysis, cell division, and nutrient uptake from the environment. Cells become infected with phages (possibly multiple times)


$$\begin{array}{c} \text{Env}[M,P]+\text{Cell}[\tau ,s,N,Q]\overset{\epsilon \;}{\to }\text{Env}[M,P-1]+\text{Cell}[\tau^{+},s,N,Q+1], \end{array}$$ (10)

where the age τ is reset upon the first infection $\tau^{+}\sim \tau$ if *Q* > 0 and zero otherwise. Once a cell is infected, it lyses with an age- and infection-dependent rate


$$\begin{array}{c} \text{Env}[M,P]+\text{Cell}[\tau ,s,N,Q]\overset{\delta (\tau ,Q)\;}{\to }\text{Env}[M,P+Q], \end{array}$$ (11)

and releases a burst of phages into the environment. Lysis occurs after a random time following a Gamma distribution φ with a fixed mean and coefficient of variation measured experimentally [67,68]. This non-Markovian infection dynamics can be implemented through an infection-age dependent lysis rate. The corresponding rate function is


$$\begin{array}{c} \delta (\tau ,Q)=\left\{ \begin{array}{cc} \varphi (\tau )/\int_{\tau }^{\infty }ds\varphi (s) & Q>0,\\ 0 & \text{otherwise}, \end{array}\right. \end{array}$$ (12)

models non-exponential infection times. These could be obtained from multi-stage infection dynamics with Erlang-distributed lysis times [64–66,69], which is a special case of the Gamma distribution. Meanwhile, uninfected cells will be able to divide


$$\begin{array}{cc} & \text{Cell}[\tau ,s,N,Q=0]\overset{\gamma (\tau ,s,\alpha )\;}{\to }\text{Cell}[0,s_{1}^{+},N_{1}^{+},0]+\text{Cell}[0,s_{2}^{+},N_{2}^{+},0], \end{array}$$ (13)

$$\begin{array}{cc} & s_{1}^{+}\sim \beta s,\;s_{2}^{+}\sim (1-\beta )s\;N_{1}^{+}\sim \text{Binomial}(\beta ,N),\;N_{2}^{+}\sim N-N_{1}^{+}, \end{array}$$ (14)

and partition intracellular nutrients between the daughter cells, similar to the gene expression model in Section 3.1. Uptake is modeled by importing nutrients from the environment into the cell:


$$\begin{array}{c} \text{Env}[M,P]+\text{Cell}[\tau ,s,N,Q]\overset{\beta \;}{\to }\text{Env}[M-1,P]+\text{Cell}[\tau ,s,N+1,Q], \end{array}$$ (15)

Intracellular nutrients are then consumed through metabolic processes fueling growth, but can also be directed towards replicating phages in infected cells:


$$\begin{array}{c} Q+N\overset{k/s\;}{\to }2Q,\;N\overset{\alpha (N)\;}{\to }\emptyset ,\;\frac{\partial s}{\partial t}=\alpha (N)s, \end{array}$$ (16)

where α(N) is the cellular growth rate following Monod’s equation.

We simulated the model starting from a single cell and a fixed number of phages in the environment (S4 Code). The cell population initially expanded, peaked, and then rapidly collapsed (Fig 4b), a pattern also observed in deterministic models [64]. Surprisingly, in our stochastic model, both the peak size and timing were highly variable (Fig 4c). Peak size was inversely related to large bursts of phages released into the environment by cell lysis events (Fig 4d). We found that the distribution of extinction times was bimodal, displaying subpopulations that experienced early and late collapse (Fig 4e). Early collapse correlated with phage bursts from a few infected cells (blue lines, Fig 4d). We therefore analyzed the burst distributions from individual lysis events in these populations that we tracked using the keyword @saveinstate (S4 Code). Burst distributions were much wider for the early-collapsing lineages, exhibiting an order-of-magnitude increase in the distribution mode. Such burst stochasticity is not accounted for in deterministic models but has been quantified experimentally [70]. Recent advances in single-cell microfluidics [71,72] provide a fine-grained picture of phage-replication dynamics, and the modeling framework presented here could facilitate integrating these new single-cell insights into agent-based models of population dynamics.

**Fig 4:**
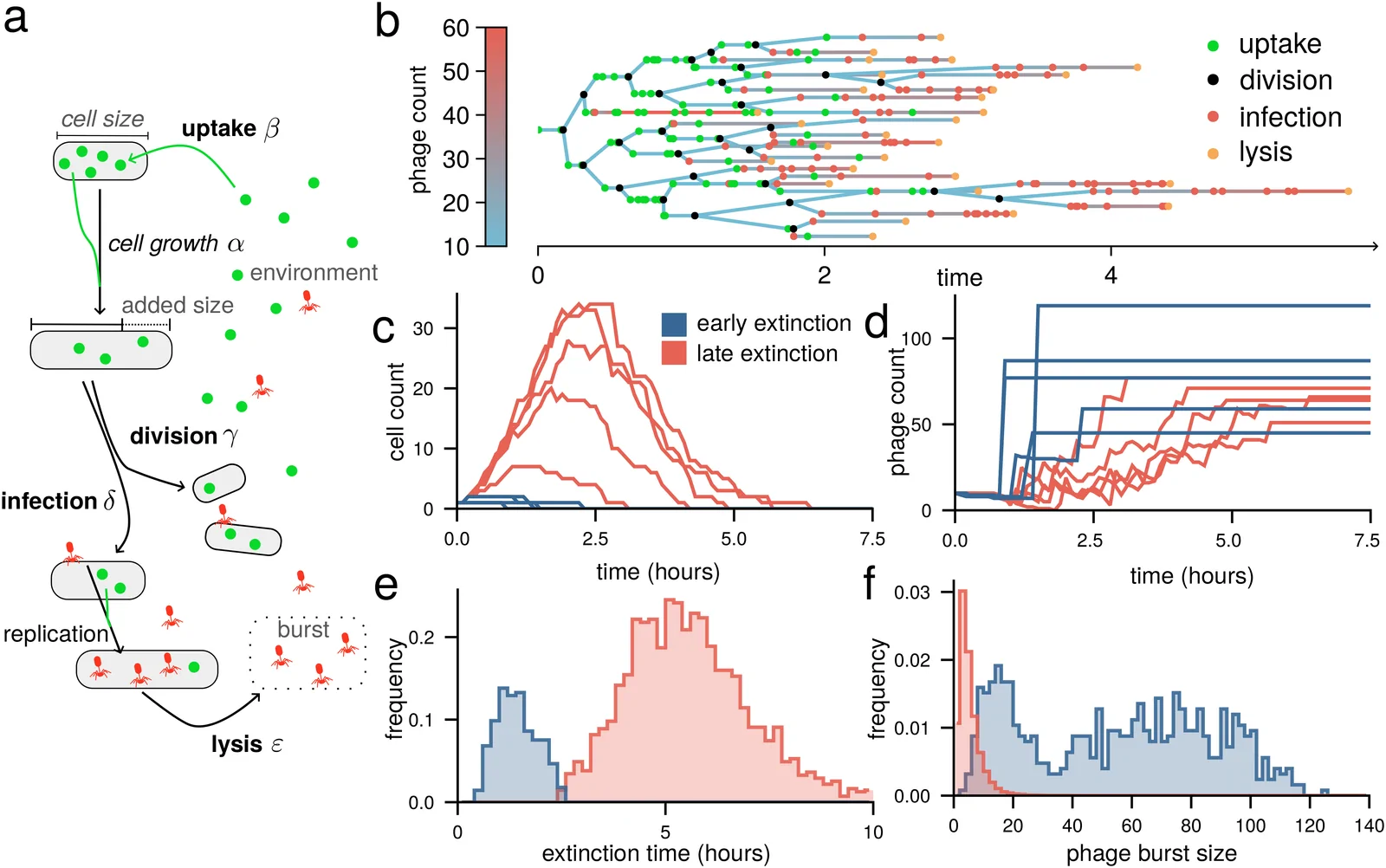
Stochastic lysis kinetics induces bimodal extinction time distributions in an agent-based phage infection model. **(a)** Model illustration of cellular agents that grow and divide after taking up nutrients from the environment. Phages infect cells and stochastically replicate inside cells in a nutrient-dependent manner, and lyse cells, releasing bursts of phages. **(b)** Lineage following cell division, nutrient uptake, infection and lysis across a micro-colony of cells. Line color represents intracellular phage levels. **(c)** Population trajectory following infection of a single cell. **(d)** Burst of phages following infection is stochastic. **(e)** Extinction times show bimodal distribution with populations divided in early and late extinction. **(f)** Phage burst distributions of early and late extinct populations.

### 3.4. Discussion of findings and extensions

Our first example — modeling stochastic gene expression in growing and dividing cells — demonstrates how gene expression can be coupled to cell growth and division processes. Cells in this model grow exponentially and divide according to an “adder” cell size control mechanism, while proteins are synthesized in bursty, size-dependent reactions. We validated the exact simulations against analytical predictions for protein distributions at birth, finding direct agreement with our implementation. While we recovered known single-cell patterns, such as size-protein distributions, our model could easily be extended to include metabolism or gene regulation. Our simulation preserves trajectories, tracking individual cells over time, enabling other lineage-tree analyses similar to those in live-cell microscopy or lineage-tracing experiments.

An alternative to the model with added size-dependent division rate, is a multi-stage process that also accounts for the same adder behavior, and these models have been used to study the effect of gene replication on mRNA and protein expression [48,73–75]. We coupled a simple model of bursty gene expression to a multi-stage model of division and replication using *AgentBasedModeling.jl* (S4 Code and Section A in S1 Appendix). The multi-stage model reproduces the same cell size distributions, but it alters the gene expression distributions (Fig A in S1 Appendix). This is because the model lacks stochastic concentration homeostasis, which also prevents it from admitting simple analytical solutions [47,48].

The second case study — cell communication — illustrates how the framework can model intercellular signaling common to a broad range of microbial and mammalian systems. Cells exchange molecules with the environment, creating a shared pool that cells respond to in a stochastic, density-dependent manner via a positive feedback mechanism. Our simulation results reveal that feedback and stochasticity can give rise to complex, emergent behavior such as the coexistence of induced and repressed states at the single-cell and population level. Notably, at low population sizes, we observed that the temporal correlation between intra- and extracellular levels increased when cells switched synchronously between global on- and off-states. These transitions are driven by intrinsic noise, which is typically ignored in mean-field models.

Our third application – bacteriophage λ infection and lysis – further illustrates how stochastic intracellular processes can determine the population fate. As cells grow and divide, phages replicate stochastically within the host. Upon reaching a critical state, infected cells lyse, releasing a burst of phages that can infect other cells. We explicitly modeled lysis timing and burst variability, which are supported by experimental measurements but typically neglected or incorporated using the linear chain trick into deterministic models [64–66,69]. Simulations revealed that the timing and magnitude of early lysis bursts determine the population trajectory. We observed a bimodal distribution of extinction times: some populations collapsed early after large initial bursts, while others grew for longer before eventually collapsing. This heterogeneity is not captured by mean-field models, which predict extinction only over infinite-time horizons as they average over burst timings and lysis events. By contrast, our agent-based framework can trace stochastic events and cell histories to identify rare, high-impact lysis events that determine population fate.

We investigated whether bimodal extinction distributions are unique to phage infections or a general feature of stochastic infection models. We developed an agent-based SIR model with infection-age-dependent rates and population heterogeneity [76], including factors like incubation periods, age structure, and vaccination status (Section B in S1 Appendix, S5 Code, Fig Ba in S1 Appendix), which have previously been studied using partial differential equations [35]. Extinction occurred either early without outbreak or after the first or second wave due to low infection numbers, leading to multimodal extinction time distributions similar to what we observed in the phage model (Fig Bb in S1 Appendix). While multimodality persisted when ignoring population structure using Gillespie simulations [77], the age-structured model predicted a lower outbreak probability and delays in extinction times of outbreaks consistent with incubation effects (Fig Bc in S1 Appendix). These findings highlight the interplay of stochasticity and population structure underpinning infection dynamics.

Together, these case studies highlight the versatility of *AgentBasedModeling.jl*. Across very different biological scenarios—intracellular gene regulation, intercellular signaling, and host-pathogen interactions—our framework provides a unified formalism of structured population dynamics through evolving internal states and stochastic interactions.

## 4. Availability and future directions

Cellular systems can be understood as heterogeneous, interacting cellular agents whose behaviors are governed by both internal biological network dynamics and external environmental cues. Capturing such complexity requires frameworks that integrate fine-grained mechanistic intracellular processes with stochastic interactions across a cell population. Here, we introduced *AgentBasedModeling.jl*, a Julia-based platform that enables efficient, flexible simulation of structured populations governed by stochastic, continuous-time agent-based models. The Julia package is freely available with open source code and usage examples at https://github.com/pihop/AgentBasedModeling.jl.

A key strength of our computational framework is its ability to represent cells as agents whose internal dynamics evolve according to jump-diffusions and interact through continuous-rate processes that respond to cell states. This enables biologically realistic modeling of systems where internal fluctuations—such as noise in gene expression, metabolism and growth, or stochastic infection and cell death directly influence population-level behavior. By combining state-of-the-art modeling tools (*ModelingToolkit.jl* and *Catalyst.jl*) with a novel event-driven simulation algorithm, *AgentBasedModeling.jl* presents a powerful and extensible tool for computational biology.

Compared to existing simulation tools often tailored to spatial models [18–20], *AgentBasedModeling.jl* offers several advantages. First, it allows continuous-time simulation with exact handling of interactions with non-exponential waiting times via thinning-based samplers. Second, it supports hybrid dynamics, including ODEs, SDEs, and discrete jumps, enabling realistic mechanistic models of intracellular behavior. Third, our tool extends Markov jump processes of single agents, such as provided by *PyEcoLib* [29] or *JumpProcesses.jl*, to populations of interacting agents. Finally, the interface is composable and integrates with the broader Julia ecosystem, supporting parameter inference [17], visualization [78], and deployment in reproducible pipelines.

We anticipate our framework to advance the state-of-the-art of computational cell models. The ability to simulate and track individual cells as agents makes it suitable for simulation-based inference [14,15], especially in settings where lineage trees, time-lapse imaging, or single-cell omics data are available. The modular design allows rapid model construction and exploration of alternative hypotheses, which is valuable for both theoretical investigations and synthetic biology applications. Our tool could further facilitate the integration of stochastic population models with existing agent-based simulation environments. Many current agent-based frameworks rely on fixed discrete update times, whereas biologically relevant events such as intracellular reactions, growth, division, death, and cell-cell interactions occur concurrently in continuous time. This mismatch can distort event timing and ordering across many interacting cells, with consequences for emergent behavior even with advanced updating schedules. Continuous-rate, event-driven formulations such as those implemented in *AgentBasedModeling.jl* offer a natural route to overcoming these limitations and to coupling mechanistic intracellular models with larger-scale stochastic population simulations. Future extensions could include explicit spatial structure, history-dependent epigenetic rules, and adaptive agent behavior, allowing more faithful models of development, immunity, and ecological systems.

*AgentBasedModeling.jl* fills a critical gap in computational biology by enabling exact agent-based simulation and easy model specification of complex, stochastic intracellular dynamics that drive emergent population behavior.

## Supporting information

S1 Appendix Supporting information describing stochastic gene expression model coupled to a multi-stage division and replication (Section A) and epidemic model of infection age with heterogeneous mixing (Section B). (PDF)

S1 Code Pluto notebook: Stochastic gene expression in growing and dividing cells. (JL)

S2 Code Pluto notebook: Cell-cell communication. (JL)

S3 Code Pluto notebook: Bacteriophage infection. (JL)

S4 Code Pluto notebook: Stochastic gene expression model coupled to a multi-stage division and replication. (JL)

S5 Code Pluto notebook: Susceptible-infected-recovered epidemic model. (JL)
