# Supplementary material for: AgentBasedModeling.jl: A tool for stochastic simulation of structured population dynamics
Source: PLoS Comput Biol. 2026 Jul 29;22(7):e1014366. doi: 10.1371/journal.pcbi.1014366 (PMC13722299; doi:10.1371/journal.pcbi.1014366)
Supplement: Supplementary file 1 [file pcbi.1014366.s001.pdf]

AgentBasedModeling.jl: a tool for stochastic simulation of structured population dynamics

Paul Piho and Philipp Thomas

Department of Mathematics, Imperial College London, London, UK.

Contributing authors: paul.piho@tuta.com; p.thomas@imperial.ac.uk;

All Pluto notebooks are available under
https://pihop.github.io/AgentBasedModeling.jl

S1 Appendix

A Stochastic gene expression coupled to a multi-stage division and replication model

We discuss here an extension of our gene expression model coupled to a multi-stage division process with gene replication (S4 Code). In the model, cells transition through N stages and duplicate their DNA at stage $M < N$. Denoting the cell state by stage n, cell size s, protein p and gene copy number d, these transitions can be written as

$$\text{Cell}[n, s, p, d] \xrightarrow{\alpha s \delta_{n<N}} \text{Cell}[n + 1, s, p, (1 + \delta_{n,M})d]. \tag{S1}$$

and cell division at the final stage

$$\text{Cell}[n, s, p, d] \xrightarrow{\alpha s \delta_{n\geq N}} \text{Cell}[0, s_1^+, p_1^+, d_1^+] + \text{Cell}[0, s_2^+, p_2^+, d_2^+], \tag{S2}$$

$$s_1^+ \sim \beta s, \quad s_2^+ \sim (1-\beta)s, \quad p_1^+ \sim \text{Binomial}(\beta, p), \quad p_2^+ \sim p - p_1^+, \quad d_{1,2}^+ = d/2, \tag{S3}$$

where the DNA content is segregated deterministically to the daughter cells.

The resulting multi-stage model can be implemented as follows:

Step 1: Stochastic gene expression coupled to a multi-stage division and replication model

```julia
using Catalyst, Distributions, AgentBasedModeling
```

```
@abm_variables C(t)
@parameters Cs CΔ Cp Cn Cdna β B L M

transitionMS = @interaction begin
    @channel γMS($Cs, $CΔ, $α, $Cn), C --> C
    @sampler ExtrandeMethod($L; boundtype=:increasing)
    @connections (($CΔ => $Δ, $Cs => $s, $Cp => $p, $Cn => $n,
        $Cdna => $dna),)
    @transition (
        ($Δ => $CΔ, $s => $Cs, $p => $Cp, $n => $Cn + 1.0, $dna
            => $Cdna + ifelse($Cn == $M, 1.0, 0.0), ),)

    @name "transition"
end

divisionMS = @interaction begin
    @channel γdivisionMS($Cs, $CΔ, $α, $Cn), C --> 2*C
    @sampler ExtrandeMethod($L; boundtype=:increasing)
    @connections (($CΔ => $Δ, $Cs => $s, $Cp => $p, $Cn => $n,
        $Cdna => $dna),)
    @variable $β = rand($Beta(100))
    @variable $B = rand($Binomial($Cp, $β))
    @transition (
        ($Δ => 0.0, $s => $β*$Cs, $p => $B, $n => 0.0, $dna =>
            $Cdna/2),
        ($Δ => 0.0, $s => (1-$β)*$Cs, $p => $Cp - $B, $n => 0.0,
            $dna => $Cdna/2))

  @name "division"
end
```

To model the effect of gene dosage on transcription, we assume that burst size scales with DNA content. This assumption leads to discontinuous changes in burst characteristics and to imperfect concentration homeostasis.

Step 2: Stochastic gene expression coupled to a multi-stage division and replication model

```
@independent_variables t
@abm_variables Δ(t) s(t) p(t) n(t) dna(t)
@parameters α kprod b
D = Differential(t)

@register_symbolic Distributions.Geometric(a)
m = rand(Distributions.Geometric(1/(1 + b*dna)))

CellDynamics = @reaction_network begin
    @species p(t) n(t) dna(t)
    @variables Δ(t) s(t)
    @equations begin
```

```
        D(s) ~ $α*s
        D(Δ) ~ $α*s
    end
    kprod, 0 --> $m*p
end

Cell = AgentDynamics(CellDynamics,());
model = AgentsModel([divisionMS, transitionMS, ], Dict(C =>
    Cell,));
```

The model simulations match the birth size distribution perfectly using the model with effective division rate (Fig Aa) in the main text (see Section *Stochastic gene expression is coupled to growth and division*). This is because the multi-stage model leads to an Erlang-distributed added size distribution [1], which is a special case of the Gamma distribution modeled by the division rate γ in the main text. However, the multi-stage model disrupts stochastic concentration homeostasis because the burst-size distribution no longer depends on cell size but on DNA content. The latter changes discretely at random times defined in the multi-stage models, which leads to a wider protein distribution (Fig Ab). Similar deviations have been studied using master equation frameworks [2, 3].

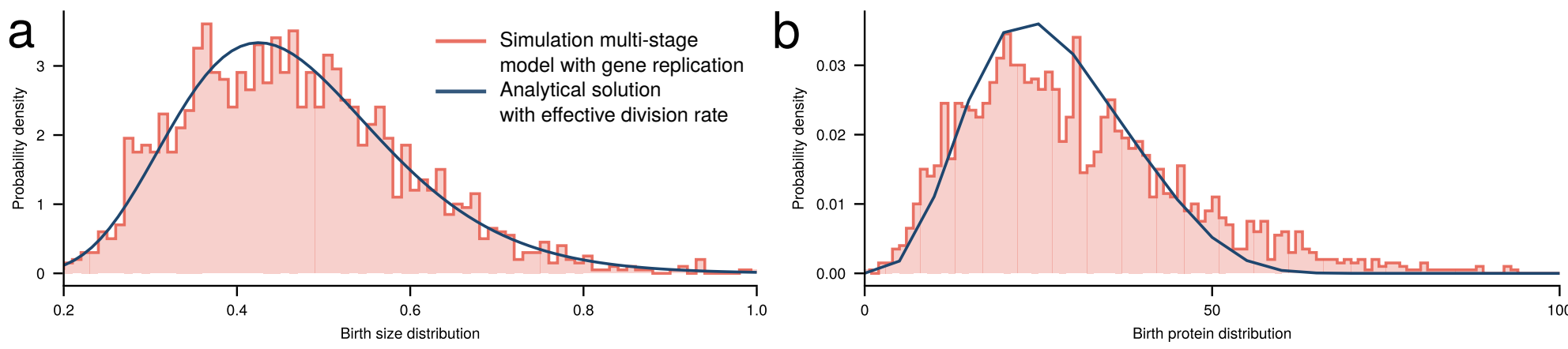

Fig A: **Stochastic gene expression with multi-stage division and gene replication. (a)** Simulation of birth size (shaded) agrees with analytical computations for the model with effective division rate (solid black). **(b)** Simulation of protein content after division (shaded) shows wider distribution than analytical solution of the effective model (solid black).

B Agent-based epidemic modeling of infection age wit heterogeneous mixing

We provide here a use case of agent-based epidemic modeling with our tool (S5 Code). We consider the stochastic SIR model (Fig Ba) to study the influence of the incubation period on the extinction probability of the disease, i.e., the epidemic burnout [4, 5, 6]. The model consists of three types of agents: susceptible (S), infected (I) and recovered (R) defined in Step 1 via the `@species` keyword. The infected agents have a continuous internal state that tracks the time since infection (τ) via a unit-rate `ODESystem`. The lack of dynamical traits in susceptible and recovered agents is handled as a `EmptyTraitProblem`. Each agent belongs to a fixed age group denoted by

`@parameters Age` drawn from a distribution of age demographics (young or old) or vaccination status (yes or no) or similar, representing additional extrinsic variability. Such models are known as age-of-infection models with heterogeneous mixing, because groups of susceptible and infected individuals shuffle throughout the outbreak, following infection and recovery dynamics [7].

Step 1: SIR model

```
@variables t
@species I(t), S(t), R(t), τ(t)
@parameters Age

D = Differential(t)
@named InfectedDyn = ODESystem([D(τ) ~ 1.0, ], t)
@named SusceptibleDyn = EmptyTraitProblem()
@named RecoveredDyn = EmptyTraitProblem()

Infected = AgentDynamics((InfectedDyn,), (Age,))
Susceptible = AgentDynamics((SusceptibleDyn,), (Age,))
Recovered = AgentDynamics((RecoveredDyn,), (Age,))
```

We first explain how the infections are modeled in Step 2. Here, a susceptible agent becomes infected through contact with an infected agent:

$$S[a_1] + I[\tau, a_2] \xrightarrow{\gamma(\tau, a_1)} I[0, a_1] + I[\tau, a_2]. \tag{S4}$$

The infection rate $\gamma(\tau, a)$ depends both on the infected agent's time since infection τ and the susceptible individual's age group a_1. The interaction is implemented as the interaction channel via the `@interaction` keyword and simulated using `@sampler ExtrandeMethod`(...). Similarly, we model recovery through an interaction with a constant rate

$$I[\tau, a] \rightarrow R[a], \tag{S5}$$

that tracks the age group. This interaction can be sampled via the standard method `@sampler GillespieMethod()`.

Step 2: SIR model: infection and recovery

```
@parameters ...
infection = @interaction begin
  @channel γ_infect($Iτ, $SAgeGroup) / N,
           $I + $S --> 2 * $I
  @sampler ExtrandeMethod($γinfectbound($Iτ, $SAgeGroup),$L)
  @connections ($Iτ, $I, $τ)
               ($SAgeGroup, $S, $AgeGroup)
               ($IAge, $I, $AgeGroup)
  @transition (
    ($τ => $Iτ, $AgeGroup => $IAgeGroup),
```

```
    ($τ => 0.0, $AgeGroup => $SAgeGroup))
end

recovery = @interaction begin
  @channel γ, $I --> $R
  @connections ($IAge, $I, $Age)
  @sampler GillespieMethod()
  @transition (($Age => $IAge, ), )
end
```

In Step 3, we consider extrinsic variability modeled through age-group-dependent immigration

$$\emptyset \xrightarrow{\mu} S[a], \qquad a \sim \pi, \tag{S6}$$

where group membership follows a categorical distribution π. Since the immigration rate is constant, it can be simulated through Gillespie's algorithm, similar to the standard emigration reactions $S/I/R \to \emptyset$.

Step 3: SIR model: migration

```
immigration = @interaction begin
  @channel $μ*N, 0 --> $S
  @variable $a_ = $sample_age()
  @transition (($τ => 0.0, $Age => $a_), )
  @sampler GillespieMethod()
end

s_emig = @interaction begin
  @channel $μ, $S --> 0
  @sampler GillespieMethod()
end
i_emig = @interaction begin
  @channel $μ, $I --> 0
  @sampler GillespieMethod()
end
r_emig = @interaction begin
  @channel $μ, $R --> 0
  @sampler GillespieMethod()
end
```

The interactions are combined into a model via a snippet in Step 4.

Step 4: Combine interactions and state dynamics

```
population_model = AgentsModel(
  [infection, recovery, immigration, s_emig, i_emig, r_emig],
  Dict(I => Infected, S => Susceptible, R => Recovered))
```

We obtained sample paths (Fig Bb) from agent-based simulations showing burnout events where the disease is stochastically eradicated. The burnout times follow a multimodal distribution with peaks corresponding to eradication after multiple epidemic waves. Comparing these distributions to an unstructured Gillespie simulation (red), we find that the presence of an incubation period increases burnout probability initially but delays burnouts after the first and second outbreak waves (Fig Bc).

Simulating a large number of pairwise interactions is time-consuming, and such computational burdens can partially be alleviated through algorithmic choices (Fig Bb inset). These difficulties are common to all agent-based frameworks and could be diminished through integration with coarse-graining methodologies such as in [8].

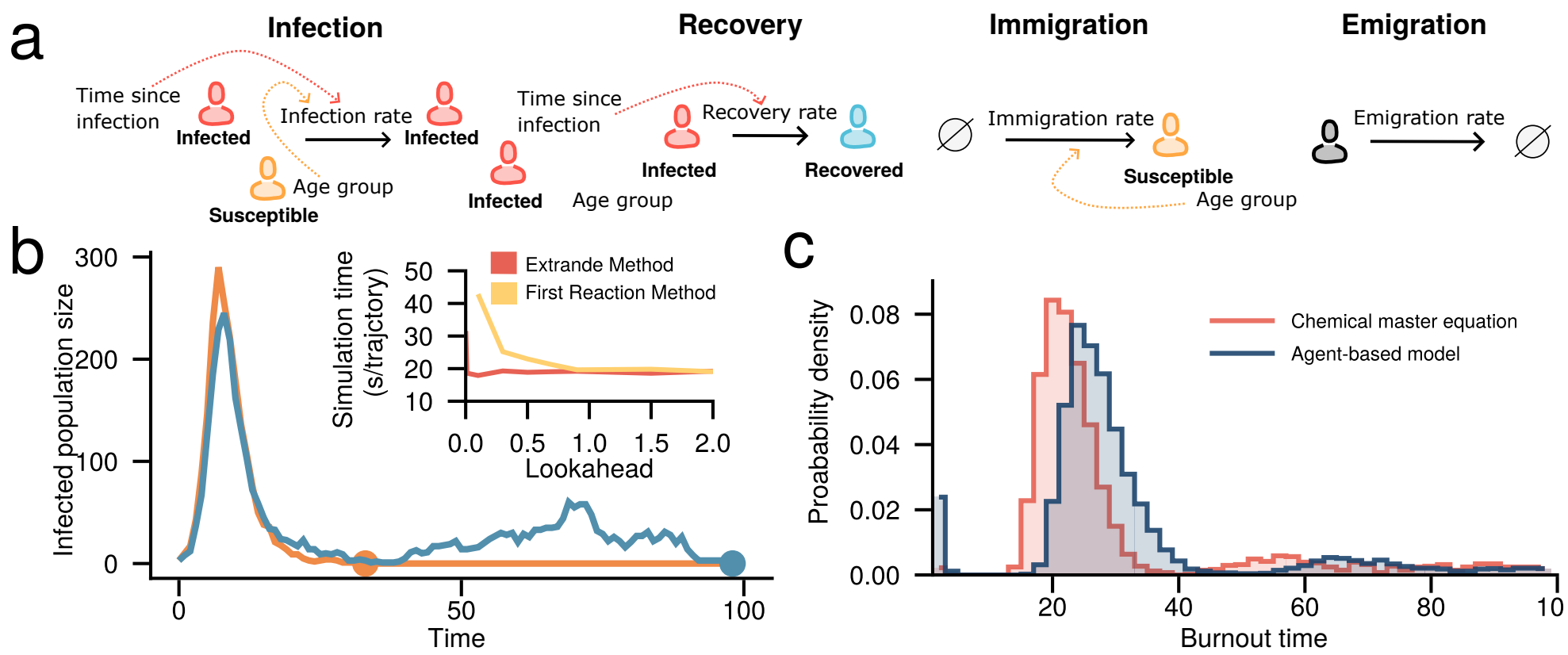

Fig B: **Agent-based epidemic modeling.** **(a)** Agent interactions of an SIR model. **(b)** Two sample trajectories of the agent-based model showing the epidemic burnout where the number of infected agents in a population becomes 0. The inset shows the dependence of the simulation time for the agent-based model on the chosen lookup horizon. **(c)** Comparison of epidemic burnout distributions for the chemical master equation and agent-based

References

[1] César Nieto, Juan Arias-Castro, Carlos Sánchez, César Vargas-García, and Juan Manuel Pedraza. "Unification of cell division control strategies through continuous rate models". *Physical Review E* 101.2 (2020), p. 022401. DOI: 10.1103/PhysRevE.101.022401.

[2] Chen Jia and Ramon Grima. "Coupling gene expression dynamics to cell size dynamics and cell cycle events: Exact and approximate solutions of the extended telegraph model". *iScience* 26 (2023). DOI: 10.1016/j.isci.2022.105746.

[3] Joseph R Peterson, John A Cole, Jingyi Fei, Taekjip Ha, and Zaida A Luthey-Schulten. "Effects of DNA replication on mRNA noise". *Proceedings of the National Academy of Sciences* 112.52 (2015), pp. 15886–15891. DOI: 10.1073/pnas.1516246112.

[4] Fred Brauer. "Age of Infection Epidemic Models". *Mathematical and Statistical Modeling for Emerging and Re-emerging Infectious Diseases*. Ed. by Gerardo Chowell and James M. Hyman. Cham: Springer International Publishing, 2016, pp. 207–220. ISBN: 978-3-319-40413-4. DOI: 10.1007/978-3-319-40413-4_13. URL: https://doi.org/10.1007/978-3-319-40413-4_13.

[5] Hisashi Inaba. *Age-Structured Population Dynamics in Demography and Epidemiology*. Singapore: Springer, 2017. DOI: 10.1007/978-981-10-0188-8.

[6] Todd L. Parsons, Benjamin M. Bolker, Jonathan Dushoff, and David J. D. Earn. "The Probability of Epidemic Burnout in the Stochastic SIR Model with Vital Dynamics". *Proceedings of the National Academy of Sciences* 121 (2024), e2313708120. DOI: 10.1073/pnas.2313708120.

[7] Fred Brauer and James Watmough. "Age of infection epidemic models with heterogeneous mixing". *Journal of Biological Dynamics* 3.2-3 (2009), pp. 324–330. DOI: 10.1080/17513750802415822.

[8] John T. Nardini, Ruth E. Baker, Matthew J. Simpson, and Kevin B. Flores. "Learning Differential Equation Models from Stochastic Agent-Based Model Simulations". *Journal of The Royal Society Interface* 18 (2021), p. 20200987. DOI: 10.1098/rsif.2020.0987.
