# Supplementary material for: AgentBasedModeling.jl: A tool for stochastic simulation of structured population dynamics
Source: PLoS Comput Biol. 2026 Jul 29;22(7):e1014366. doi: 10.1371/journal.pcbi.1014366 (PMC13722299; doi:10.1371/journal.pcbi.1014366)
Supplement: Supplementary file 2 [file pcbi.1014366.s007.pdf]

Response to reviewers (PCOMPBIOL-D-25-01869)

Journal Requirements:

1) We ask that a manuscript source file is provided at Revision. Please upload your manuscript file as a .doc, .docx, .rtf or .tex. If you are providing a .tex file, please upload it under the item type u2018LaTeX Source Fileu2019 and leave your .pdf version as the item type u2018Manuscriptu2019.

2) Your manuscript is missing the following sections: Design and Implementation, Results, and Availability and Future Directions. Please ensure that your article adheres to the standard Software article layout and order of Abstract, Introduction, Design and Implementation, Results, and Availability and Future Directions. For details on what each section should contain, see our Software article guidelines:

https://journals.plos.org/ploscompbiol/s/submission-guidelines#loc-software-submissions

3) Please upload all main figures as separate Figure files in .tif or .eps format. For more information about how to convert and format your figure files please see our guidelines:
https://journals.plos.org/ploscompbiol/s/figures

4) We have noticed that you have a list of Supporting Information legends in your manuscript. However, there are no corresponding files uploaded to the submission. Please upload them as separate files with the item type 'Supporting Information'.

5) We notice that your supplementary information is included in the manuscript file. Please remove them and upload them with the file type 'Supporting Information'. Please ensure that each Supporting Information file has a legend listed in the manuscript after the references list.

6) Please amend your detailed Financial Disclosure statement. This is published with the article. It must therefore be completed in full sentences and contain the exact wording you wish to be published.
- State the initials, alongside each funding source, of each author to receive each grant. For example: "This work was supported by the National Institutes of Health (####### to AM; ###### to CJ) and the National Science Foundation (###### to AM)."
- State what role the funders took in the study. If the funders had no role in your study, please state: "The funders had no role in study design, data collection and analysis, decision to publish, or preparation of the manuscript.".
If you did not receive any funding for this study, please simply state: u201cThe authors received no specific funding for this work.u201d

We have now formatted our manuscript according to the journal requirements, including section names, supporting information files. All changes have been highlighted in magenta.

Reviewer #1:

In this paper, the authors (Piho and Thomas) proposed a novel computational package for cell populations involving intracellular dynamics, cell division, cell-cell interactions, etc. The details of the package are clearly explained and demonstrated through several numerical examples.

In general, this paper is clearly written, and the package is useful for cell biological studies. The manuscript can be further improved by making the following points clearer.

We thank the reviewer for the positive assessment of our manuscript. We have addressed all comments below.

— Eq. (2) and the interaction rules are not so clear to me. I can roughly understand that the interaction mechanisms could remove certain agents from the system and produce new ones. However, it is unclear what are removed and produced. This is particularly because the definition of S[x] is not given.

We apologize for the lack of clarity in the previous manuscript. S[x] denotes the agents with internal states x. Input agents with specific states x^- are removed, and output agents are initialized with new states x^+. Although the dependence on the internal states is slightly verbose, it serves to associate agents with their corresponding states, as the interaction rate is computed. We have updated the notation and included additional biological examples to clarify the use of these interaction rules.

— Also, the difference between agents and agent types is confusing. It should be made clear whether Eq. (1) represents the dynamics of one agent of type S_j, or the j-th agent. If it is the former case, then the noise term W_j and Y_{r,j} should contain another notation to indicate that different agents are corrupted with different noise.

The reviewer is correct about the confusion. We have now introduced index (i) for the agent, distinguishing the agent from its type (index j).

Reviewer #2:

The manuscript “AgentBasedModeling.jl: a tool for stochastic simulation of structured population Dynamics” presents an agent based simulation tool that incorporates the stochasticity at both the single cell and population levels. The approach is technically correct and the applicability is wide. I have some observations on the setup for the examples of use, but in general I would recommend for publication with minor changes.

Main comments:
As our understanding of gene noise and its phenotypic consequences at the individual cell level deepens, and the importance of this at the population dynamics level becomes clearer, the need for simulation tools that allow for the exploration of this interplay becomes apparent. Previous tools such as SGNS2 and PyEcoLib have incorporated the stochasticity at the individual cell level, but are trickier to incorporate in a population level simulation. AgentBasedModeling.jl is a simulation tool that natively takes into account the interplay of the stochastic processes at both scales, so I believe it to be a useful, novel contribution.

We thank the reviewer for the positive assessment of our manuscript. We have addressed all comments below.

Where I would suggest some changes is in the setup for the examples of use. In particular, in section 2.5.1 the authors simulate growth via an adder mechanism. This is a phenomenological model, not applicable in all cases, and is an emergent property which comes about as a statistical consequence of a specific underlying stochastic process. In particular, if the probability of division is proportional to the current size (with an exponent to include cases that are sizer-like or timer-like rather than pure adder) the adder effect comes out naturally. I mention this because this is precisely the kind of situation where an agent based stochastic model can naturally model the underlying process, so it seems wrong to use the emergent statistics as part of the base model rather than the stochastic process, as it requires a separate distribution of added volume that is independent of the cell state. It would in itself be a simple test/demonstration of the tool to have the adder come out of the cell state description.

The reviewer is raising an important point. We used the phenomenological process since the distribution of added size could be measured directly from experiments. The advantage of the underlying multi-stage stochastic process is that it is readily interpretable and straightforward to implement and extend using our tool. We have now included an entire new supplementary text detailing the implementation of the multi-stage model (Supporting Notebook S4 and Text S5), and we show that its simulations agree with our phenomenological model (new Supplementary Figure 1a). Furthermore, following the suggestion by Reviewer 3, we have extended this multi-stage model to account for gene-dosage effects on protein distributions due to replication (new Supplementary Figure 1b). These effects were not accounted for in our phenomenological model, and they are now discussed in the new paragraph in Section 3.4.

Minor comments:

In the author summary the phrase “watch how their interactions give rise to collective behavior” seems more informal than the rest of the text

Thank you. We have changed the wording.

I would expand on the difficulties arising from the mismatch between stochastic timings when trying to use existing ABM tools within a larger stochastic population simulation. Many people don´t have an intuition for simulations without predefined time steps and might not realize the issue.

Thank you for the suggestion. We have expanded on this issue in the final section 4, *Availability and Future Work*.

Reviewer #3:

The manuscript "AgentBasedModeling.jl: a tool for stochastic simulation of structured population dynamics" presents a very general framework that integrates intracellular state dynamics with population-level interactions, allowing a wide range of biological models to be examined within a unified stochastic simulation framework. Overall, the structure of the manuscript is clear, and the proposed framework is well motivated and technically sound. The examples effectively demonstrate the flexibility and potential usefulness of the tool. While the work

is valuable and generally well presented, several issues still need attention. I would strongly recommend publication once these issues have been addressed.

We thank the reviewer for the positive assessment of our manuscript.

1. The notation system used in the manuscript is somewhat confusing and would benefit from clarification. For example, in Equation (2), the indices of the agent type labels and the corresponding state vectors are written in the same way, which may cause ambiguity. When considering processes such as cell division (e.g., Equation (3)), the two daughter cells generally have different state vectors, yet the notation inherited from Equation (2) may give the impression that the daughter cells share identical states.

We thank the reviewer for pointing out that the notation was ambiguous. We have now rewritten Equation (3) to show the correct index of state vectors in the interaction rule.

In Equation (7), both the extracellular and intracellular inducer molecules are denoted by x, which makes them difficult to distinguish. I recommend revising the notation to more clearly distinguish cell identities from their associated state vectors.

We have now revised the notation around Eq. (7), labeling y as the extracellular inducer molecules.

2. There are numerous typos throughout the manuscript that should be corrected to avoid confusion. In particular, the following issues need attention:

In Equation (1), $\sigma(x_j(s))$ should be $\sigma(x_j(t))$;

The caption for Figure 2 is missing descriptions for panels (e) and (f);

In Figure 3, the legend colors in panel (b) are reversed;

In Section 2.5.3, the symbols for intracellular and extracellular nutrients and phages are incorrect;

In Equation (10), the meaning of $\tau^+\sim\tau$ is unclear and should be clarified;

In Equation (12), the age τ of the agent participating in the reaction should be 0;

In Equation (17), $S[a]$ should be $R[a]$ since the SIR model is considered here rather than the SIS model;

On Page 25, third line from the bottom, "Fig.5d" should be "Fig.5b".

We appreciate the reviewer pointing out these typos. We fixed all typos in the revised manuscript.

3. The three sampling methods described in the manuscript are all based on Markovian dynamics. However, in Equation (11), the lysis time of an infected cell is assumed to follow a gamma distribution, which violates the Markov property. This inconsistency should be addressed or clarified.

We thank the reviewer for this comment. In Section 2.3, we now clarify that state-dependent rates can induce an effective time dependence of the rates, resulting in non-exponential waiting times, even if the underlying full process remains Markovian in an expanded state space. This is a natural embedding of non-Markovian dynamics within a Markovian framework with an extended state space. For example, in Equation (11), when the lysis rate depends on the time since infection, it can be modeled by Equation (12) to capture any desired waiting-time distribution. If, on the other hand, the lysis rate is constant, i.e., independent of the time of infection, the waiting times are exponential and memoryless. We added a remark at the end of Section 2.3 and a line before Equation (12) to highlight this point.

4. The framework introduced at the beginning of the manuscript is quite abstract. I suggest adding 1-2 illustrative examples in Section 2.1 to help readers better understand the concepts. For instance, it would be helpful to clarify what an "agent" typically represents, how "agent types" are defined, and what is meant by a "channel" in this context.

We have introduced a new paragraph at the end of Section 2.1 that explains the meaning of an interaction channel and provides illustrative examples of interaction channels among cell populations.

5. After gene replication, the gene copy number increases from 1 to 2 in haploid cells (and from 2 to 4 in diploid cells). As a result, the gene expression dynamics may change substantially, since two gene copies are actively transcribed after replication, whereas only one is active before replication. Several modeling studies have explicitly incorporated this effect, such as

- Coupling gene expression dynamics to cell size dynamics and cell cycle events: exact and approximate solutions of the extended telegraph model, iScience 26(1):105746, 2023;
- Frequency domain analysis of fluctuations of mRNA and protein copy numbers within a cell lineage: theory and experimental validation, Physical Review X 11(2):021032, 2021.

Could the current modeling framework be extended to account for this important phenomenon?

We thank the reviewer for the suggestion. We have added the new Supplementary Text S5 that illustrates a multi-stage division and replication coupled to gene expression. We find that the distributions of protein but not cell size differ from our effective rate model (Supplement Fig. S1). This is due to the lack of concentration homeostasis caused by the gene-dosage effect, which we now discuss (see new paragraph in Section 3.4) and cite the suggested references.
