# Supplementary material for: AgentBasedModeling.jl: A tool for stochastic simulation of structured population dynamics
Source: PLoS Comput Biol. 2026 Jul 29;22(7):e1014366. doi: 10.1371/journal.pcbi.1014366 (PMC13722299; doi:10.1371/journal.pcbi.1014366)
Supplement: Peer review history

# Peer review: Decision Letter 0

**DOI:** 10.1371/journal.pcbi.1014366.r001

**Authors:** Qing Nie, Marc R Birtwistle

25 Nov 2025

AgentBasedModeling.jl: a tool for stochastic simulation of structured population dynamics

PLOS Computational Biology

Dear Dr. Thomas,

Thank you for submitting your manuscript to PLOS Computational Biology. After careful consideration, we feel that it has merit but does not fully meet PLOS Computational Biology's publication criteria as it currently stands. Therefore, we invite you to submit a revised version of the manuscript that addresses the points raised during the review process.

Please submit your revised manuscript by Jan 25 2026 11:59PM. If you will need more time than this to complete your revisions, please reply to this message or contact the journal office at ploscompbiol@plos.org. When you're ready to submit your revision, log on to https://www.editorialmanager.com/pcompbiol/ and select the 'Submissions Needing Revision' folder to locate your manuscript file.

Please include the following items when submitting your revised manuscript:

* A rebuttal letter that responds to each point raised by the editor and reviewer(s). You should upload this letter as a separate file labeled 'Response to Reviewers'. This file does not need to include responses to formatting updates and technical items listed in the 'Journal Requirements' section below.

* A marked-up copy of your manuscript that highlights changes made to the original version. You should upload this as a separate file labeled 'Revised Manuscript with Track Changes'.

* An unmarked version of your revised paper without tracked changes. You should upload this as a separate file labeled 'Manuscript'.

If you would like to make changes to your financial disclosure, competing interests statement, or data availability statement, please make these updates within the submission form at the time of resubmission. Guidelines for resubmitting your figure files are available below the reviewer comments at the end of this letter

We look forward to receiving your revised manuscript.

Kind regards,

Qing Nie

Academic Editor

PLOS Computational Biology

Marc Birtwistle

Section Editor

PLOS Computational Biology

**Journal Requirements:**

1) We ask that a manuscript source file is provided at Revision. Please upload your manuscript file as a .doc, .docx, .rtf or .tex. If you are providing a .tex file, please upload it under the item type u2018LaTeX Source Fileu2019 and leave your .pdf version as the item type u2018Manuscriptu2019.

2) Your manuscript is missing the following sections: Design and Implementation, Results, and Availability and Future Directions. Please ensure that your article adheres to the standard Software article layout and order of Abstract, Introduction, Design and Implementation, Results, and Availability and Future Directions. For details on what each section should contain, see our Software article guidelines:

https://journals.plos.org/ploscompbiol/s/submission-guidelines#loc-software-submissions

3) Please upload all main figures as separate Figure files in .tif or .eps format. For more information about how to convert and format your figure files please see our guidelines:

https://journals.plos.org/ploscompbiol/s/figures

4) We have noticed that you have a list of Supporting Information legends in your manuscript. However, there are no corresponding files uploaded to the submission. Please upload them as separate files with the item type 'Supporting Information'.

5) We notice that your supplementary information is included in the manuscript file. Please remove them and upload them with the file type 'Supporting Information'. Please ensure that each Supporting Information file has a legend listed in the manuscript after the references list.

6) Please amend your detailed Financial Disclosure statement. This is published with the article. It must therefore be completed in full sentences and contain the exact wording you wish to be published.

- State the initials, alongside each funding source, of each author to receive each grant. For example: "This work was supported by the National Institutes of Health (####### to AM; ###### to CJ) and the National Science Foundation (###### to AM)."

- State what role the funders took in the study. If the funders had no role in your study, please state: "The funders had no role in study design, data collection and analysis, decision to publish, or preparation of the manuscript.".

If you did not receive any funding for this study, please simply state: u201cThe authors received no specific funding for this work.u201d

**Reviewers' comments:**

Reviewer's Responses to Questions

**Comments to the Authors:**

**Please note here if the review is uploaded as an attachment.**

Reviewer #1: In this paper, the authors (Piho and Thomas) proposed a novel computational package for cell populations involving intracellular dynamics, cell division, cell-cell interactions, etc. The details of the package are clearly explained and demonstrated through several numerical examples.

In general, this paper is clearly written, and the package is useful for cell biological studies. The manuscript can be further improved by making the following points clearer.

— Eq. (2) and the interaction rules are not so clear to me. I can roughly understand that the interaction mechanisms could remove certain agents from the system and produce new ones. However, it is unclear what are removed and produced. This is particularly because the definition of S[x] is not given.

— Also, the difference between agents and agent types is confusing. It should be made clear whether Eq. (1) represents the dynamics of one agent of type S_j, or the j-th agent. If it is the former case, then the noise term W_j and Y_{r,j} should contain another notation to indicate that different agents are corrupted with different noise.

Reviewer #2: The manuscript “AgentBasedModeling.jl: a tool for stochastic simulation of structured population Dynamics” presents an agent based simulation tool that incorporates the stochasticity at both the single cell and population levels. The approach is technically correct and the applicability is wide. I have some observations on the setup for the examples of use, but in general I would recommend for publication with minor changes.

Main comments:

As our understanding of gene noise and its phenotypic consequences at the individual cell level deepens, and the importance of this at the population dynamics level becomes clearer, the need for simulation tools that allow for the exploration of this interplay becomes apparent. Previous tools such as SGNS2 and PyEcoLib have incorporated the stochasticity at the individual cell level, but are trickier to incorporate in a population level simulation. AgentBasedModeling.jl is a simulation tool that natively takes into account the interplay of the stochastic processes at both scales, so I believe it to be a useful, novel contribution.

Where I would suggest some changes is in the setup for the examples of use. In particular, in section 2.5.1 the authors simulate growth via an adder mechanism. This is a phenomenological model, not applicable in all cases, and is an emergent property which comes about as a statistical consequence of a specific underlying stochastic process. In particular, if the probability of division is proportional to the current size (with an exponent to include cases that are sizer-like or timer-like rather than pure adder) the adder effect comes out naturally. I mention this because this is precisely the kind of situation where an agent based stochastic model can naturally model the underlying process, so it seems wrong to use the emergent statistics as part of the base model rather than the stochastic process, as it requires a separate distribution of added volume that is independent of the cell state. It would in itself be a simple test/demonstration of the tool to have the adder come out of the cell state description.

Minor comments:

In the author summary the phrase “watch how their interactions give rise to collective behavior” seems more informal than the rest of the text

I would expand on the difficulties arising from the mismatch between stochastic timings when trying to use existing ABM tools within a larger stochastic population simulation. Many people don´t have an intuition for simulations without predefined time steps and might not realize the issue.

Reviewer #3: The manuscript "AgentBasedModeling.jl: a tool for stochastic simulation of structured population dynamics" presents a very general framework that integrates intracellular state dynamics with population-level interactions, allowing a wide range of biological models to be examined within a unified stochastic simulation framework. Overall, the structure of the manuscript is clear, and the proposed framework is well motivated and technically sound. The examples effectively demonstrate the flexibility and potential usefulness of the tool. While the work is valuable and generally well presented, several issues still need attention. I would strongly recommend publication once these issues have been addressed.

1. The notation system used in the manuscript is somewhat confusing and would benefit from clarification. For example, in Equation (2), the indices of the agent type labels and the corresponding state vectors are written in the same way, which may cause ambiguity. When considering processes such as cell division (e.g., Equation (3)), the two daughter cells generally have different state vectors, yet the notation inherited from Equation (2) may give the impression that the daughter cells share identical states. In Equation (7), both the extracellular and intracellular inducer molecules are denoted by $x$, which makes them difficult to distinguish. I recommend revising the notation to more clearly distinguish cell identities from their associated state vectors.

2. There are numerous typos throughout the manuscript that should be corrected to avoid confusion. In particular, the following issues need attention:

In Equation (1), $\sigma(x_j(s))$ should be $\sigma(x_j(t))$;

The caption for Figure 2 is missing descriptions for panels (e) and (f);

In Figure 3, the legend colors in panel (b) are reversed;

In Section 2.5.3, the symbols for intracellular and extracellular nutrients and phages are incorrect;

In Equation (10), the meaning of $\tau^+\sim\tau$ is unclear and should be clarified;

In Equation (12), the age $\tau$ of the agent participating in the reaction should be $0$;

In Equation (17), $S[a]$ should be $R[a]$ since the SIR model is considered here rather than the SIS model;

On Page 25, third line from the bottom, "Fig.5d" should be "Fig.5b".

3. The three sampling methods described in the manuscript are all based on Markovian dynamics. However, in Equation (11), the lysis time of an infected cell is assumed to follow a gamma distribution, which violates the Markov property. This inconsistency should be addressed or clarified.

4. The framework introduced at the beginning of the manuscript is quite abstract. I suggest adding 1-2 illustrative examples in Section 2.1 to help readers better understand the concepts. For instance, it would be helpful to clarify what an "agent" typically represents, how "agent types" are defined, and what is meant by a "channel" in this context.

5. After gene replication, the gene copy number increases from 1 to 2 in haploid cells (and from 2 to 4 in diploid cells). As a result, the gene expression dynamics may change substantially, since two gene copies are actively transcribed after replication, whereas only one is active before replication. Several modeling studies have explicitly incorporated this effect, such as

- Coupling gene expression dynamics to cell size dynamics and cell cycle events: exact and approximate solutions of the extended telegraph model, iScience 26(1):105746, 2023;

- Frequency domain analysis of fluctuations of mRNA and protein copy numbers within a cell lineage: theory and experimental validation, Physical Review X 11(2):021032, 2021.

Could the current modeling framework be extended to account for this important phenomenon?

**********

**Have the authors made all data and (if applicable) computational code underlying the findings in their manuscript fully available?**

The PLOS Data policy requires authors to make all data and code underlying the findings described in their manuscript fully available without restriction, with rare exception (please refer to the Data Availability Statement in the manuscript PDF file). The data and code should be provided as part of the manuscript or its supporting information, or deposited to a public repository. For example, in addition to summary statistics, the data points behind means, medians and variance measures should be available. If there are restrictions on publicly sharing data or code —e.g. participant privacy or use of data from a third party—those must be specified.

Reviewer #1: Yes

Reviewer #2: Yes

Reviewer #3: None

**********

PLOS authors have the option to publish the peer review history of their article (what does this mean?). If published, this will include your full peer review and any attached files.

If you choose “no”, your identity will remain anonymous but your review may still be made public.

**Do you want your identity to be public for this peer review?**  For information about this choice, including consent withdrawal, please see our Privacy Policy.

Reviewer #1: No

Reviewer #2: No

Reviewer #3: No

[NOTE: If reviewer comments were submitted as an attachment file, they will be attached to this email and accessible via the submission site. Please log into your account, locate the manuscript record, and check for the action link "View Attachments". If this link does not appear, there are no attachment files.]

**Figure resubmission:**

After uploading your figures to PLOS’s NAAS tool - https://ngplosjournals.pagemajik.ai/artanalysis, NAAS will process the files provided and display the results in the "Uploaded Files" section of the page as the processing is complete. If the uploaded figures meet our requirements (or NAAS is able to fix the files to meet our requirements), the figure will be marked as "fixed" above. If NAAS is unable to fix the files, a red "failed" label will appear above. When NAAS has confirmed that the figure files meet our requirements, please download the file via the download option, and include these NAAS processed figure files when submitting your revised manuscript.

**Reproducibility:**

?>

# Author comment: Author response to Decision Letter 1

**DOI:** 10.1371/journal.pcbi.1014366.r002

12 May 2026

Attachment Submitted filename: PCB_ Response to Reviewers .pdf

# Peer review: Decision Letter 1

**DOI:** 10.1371/journal.pcbi.1014366.r003

**Authors:** Qing Nie, Marc R Birtwistle

27 May 2026

Dear Dr Thomas,

We are pleased to inform you that your manuscript 'AgentBasedModeling.jl: a tool for stochastic simulation of structured population dynamics' has been provisionally accepted for publication in PLOS Computational Biology.

Before your manuscript can be formally accepted you will need to complete some formatting changes, which you will receive in a follow up email. A member of our team will be in touch with a set of requests.

Please note that your manuscript will not be scheduled for publication until you have made the required changes, so a swift response is appreciated.

IMPORTANT: The editorial review process is now complete. PLOS will only permit corrections to spelling, formatting or significant scientific errors from this point onwards. Requests for major changes, or any which affect the scientific understanding of your work, will cause delays to the publication date of your manuscript.

Should you, your institution's press office or the journal office choose to press release your paper, you will automatically be opted out of early publication. We ask that you notify us now if you or your institution is planning to press release the article. All press must be co-ordinated with PLOS.

Thank you again for supporting Open Access publishing; we are looking forward to publishing your work in PLOS Computational Biology.

Best regards,

Qing Nie

Academic Editor

PLOS Computational Biology

Marc Birtwistle

Section Editor

PLOS Computational Biology

***********************************************************

Reviewer's Responses to Questions

**Comments to the Authors:**

**Please note here if the review is uploaded as an attachment.**

Reviewer #1: The authors have addressed all my concerns.

Reviewer #3: The authors have satisfactorily addressed my previous comments, as well as those raised by Reviewer #2. I therefore strongly recommend this paper for publication in PCB.

**********

**Have the authors made all data and (if applicable) computational code underlying the findings in their manuscript fully available?**

The PLOS Data policy requires authors to make all data and code underlying the findings described in their manuscript fully available without restriction, with rare exception (please refer to the Data Availability Statement in the manuscript PDF file). The data and code should be provided as part of the manuscript or its supporting information, or deposited to a public repository. For example, in addition to summary statistics, the data points behind means, medians and variance measures should be available. If there are restrictions on publicly sharing data or code —e.g. participant privacy or use of data from a third party—those must be specified.

Reviewer #1: Yes

Reviewer #3: None

**********

PLOS authors have the option to publish the peer review history of their article (what does this mean?). If published, this will include your full peer review and any attached files.

If you choose “no”, your identity will remain anonymous but your review may still be made public.

**Do you want your identity to be public for this peer review?**  For information about this choice, including consent withdrawal, please see our Privacy Policy.

Reviewer #1: No

Reviewer #3: **Yes:**  Chen Jia

# Editor report: Acceptance letter

**DOI:** 10.1371/journal.pcbi.1014366.r004

**Authors:** Qing Nie, Marc R Birtwistle

PCOMPBIOL-D-25-01869R1

AgentBasedModeling.jl: a tool for stochastic simulation of structured population dynamics

Dear Dr Thomas,

I am pleased to inform you that your manuscript has been formally accepted for publication in PLOS Computational Biology. Your manuscript is now with our production department and you will be notified of the publication date in due course.

The corresponding author will soon be receiving a typeset proof for review, to ensure errors have not been introduced during production. Please review the PDF proof of your manuscript carefully, as this is the last chance to correct any errors. Please note that major changes, or those which affect the scientific understanding of the work, will likely cause delays to the publication date of your manuscript.

Soon after your final files are uploaded, unless you have opted out, the early version of your manuscript will be published online. The date of the early version will be your article's publication date. The final article will be published to the same URL, and all versions of the paper will be accessible to readers.

For Research, Software, and Methods articles, you will receive an invoice from PLOS for your publication fee after your manuscript has reached the completed accept phase. If you receive an email requesting payment before acceptance or for any other service, this may be a phishing scheme. Learn how to identify phishing emails and protect your accounts at https://explore.plos.org/phishing.

Thank you again for supporting PLOS Computational Biology and open-access publishing. We are looking forward to publishing your work!

With kind regards,

Kannan R K Kuppusamy, B.TECH BIOTECHNOLOGY

PLOS Computational Biology | Carlyle House, Carlyle Road, Cambridge CB4 3DN | United Kingdom ploscompbiol@plos.org | Phone +44 (0) 1223-442824 | ploscompbiol.org | @PLOSCompBiol
